# A *Plasmodium* apicoplast-targeted unique exonuclease/FEN exhibits interspecies functional differences attributable to an insertion that alters DNA-binding

**DOI:** 10.1093/nar/gkae512

**Published:** 2024-06-18

**Authors:** Tribeni Chatterjee, Anupama Tiwari, Ritika Gupta, Himadri Shukla, Aastha Varshney, Satish Mishra, Saman Habib

**Affiliations:** Division of Biochemistry and Structural Biology, CSIR-Central Drug Research Institute, Lucknow, India; Division of Biochemistry and Structural Biology, CSIR-Central Drug Research Institute, Lucknow, India; Division of Biochemistry and Structural Biology, CSIR-Central Drug Research Institute, Lucknow, India; Division of Molecular Microbiology and Immunology, CSIR-Central Drug Research Institute, Lucknow, India; Division of Molecular Microbiology and Immunology, CSIR-Central Drug Research Institute, Lucknow, India; Division of Molecular Microbiology and Immunology, CSIR-Central Drug Research Institute, Lucknow, India; Division of Biochemistry and Structural Biology, CSIR-Central Drug Research Institute, Lucknow, India

## Abstract

The human malaria parasite *Plasmodium falciparum* genome is among the most A + T rich, with low complexity regions (LCRs) inserted in coding sequences including those for proteins targeted to its essential relict plastid (apicoplast). Replication of the apicoplast genome (plDNA), mediated by the atypical multifunctional DNA polymerase *Pf*Prex, would require additional enzymatic functions for lagging strand processing. We identified an apicoplast-targeted, [4Fe–4S]-containing, FEN/Exo (*Pf*Exo) with a long LCR insertion and detected its interaction with *Pf*Prex. Distinct from other known exonucleases across organisms, *Pf*Exo recognized a wide substrate range; it hydrolyzed 5′-flaps, processed dsDNA as a 5′-3′ exonuclease, and was a bipolar nuclease on ssDNA and RNA–DNA hybrids. Comparison with the rodent *P. berghei* ortholog *Pb*Exo, which lacked the insertion and [4Fe–4S], revealed interspecies functional differences. The insertion-deleted *Pf*ExoΔins behaved like *Pb*Exo with a limited substrate repertoire because of compromised DNA binding. Introduction of the *Pf*Exo insertion into *Pb*Exo led to gain of activities that the latter initially lacked. Knockout of *Pb*Exo indicated essentiality of the enzyme for survival. Our results demonstrate the presence of a novel apicoplast exonuclease with a functional LCR that diversifies substrate recognition, and identify it as the candidate flap-endonuclease and RNaseH required for plDNA replication and maintenance.

## Graphical Abstract

**Graphical Abstract:**
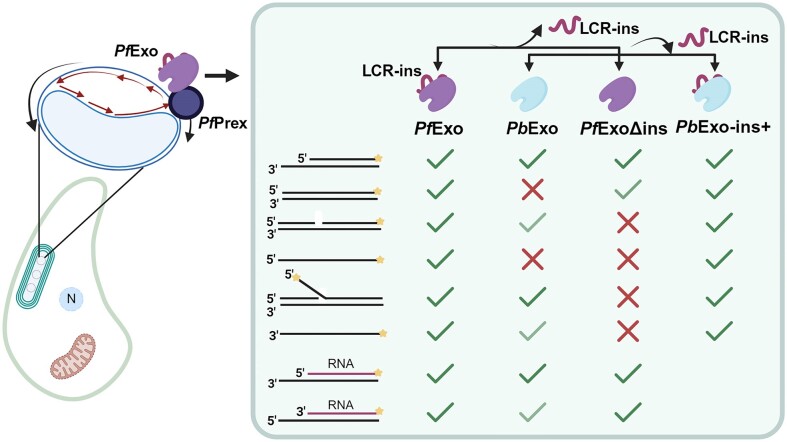

## Introduction

*Plasmodium* species, the causal agents of malaria, have two genome-containing organelles—an apicoplast (a non-photosynthetic relict plastid) and a mitochondrion. Both organelles are essential for parasite survival in the different stages of its life cycle and are considered promising targets for new drugs (1–3). The *Plasmodium falciparum* genome is A + T rich and its apicoplast genome (plDNA) is among the most A + T-rich genomes (86.9% A/T) sequenced yet. An especially high frequency of *P. falciparum* proteins, including those targeted to the apicoplast, contain >40 amino acid insertions in the form of low complexity regions (LCRs) that often form disordered domains (4). The function of these LCRs is unknown in most cases although several hypotheses have been proposed towards their role in the host immunogenic response (5–7), as tRNA sponges to help in co-translational protein folding in the parasite (8), or as recombination hotspots contributing to the generation of diversity in antigenic loci (9).

*P. falciparum* replicates via schizogony/sporogony, where segmentation of daughter cells takes place only after the final round of asynchronous nuclear division (10,11). During schizogony, the elongated and branched apicoplast undergoes fission to generate multiple daughter organelles that are packaged into daughter cells, each cell containing an apicoplast (11,12). As the parasite traverses and replicates in diverse cell types and conditions in the host and vector, maintenance of genome integrity of the replicating apicoplast 35 kb circular genome would presumably be of essence.

PlDNA is condensed by a nuclear-encoded bacterial histone-like protein *Pf*HU (13) and a single-stranded DNA binding protein *Pf*SSB helps stabilize single-stranded DNA and modify plDNA secondary structures (14). Replication of plDNA proceeds via a bi-directional *ori*/D-loop mechanism initiating at *ori* within its inverted repeat region (15,16). It is driven by a large multi-domain *Pf*Prex that consists of DNA polymerase as well as helicase and primase which are proteolytically matured (17). *Pf*Prex has proofreading 3′-5′ exonuclease activity in its Exo/Pol domain (18,19). *Pf*Prex Exo/Pol can misincorporate ribonucleotides and partially proofread them (20) and is also capable of translesion DNA synthesis (21,22). The sole DNA ligase encoded by the parasite nuclear genome is likely to function in apicoplast DNA replication/repair (23). PlDNA replication also involves a prokaryote-type topoisomerase II (DNA gyrase) (24,25) which is the major target for anti-malarial activity of fluoroquinolones and coumarins (24,26). There is a gap in knowledge about other proteins in plDNA replication in *P. falciparum* and how they might interface with DNA repair.

Base excision repair (BER) is the primary mechanism for repair of oxidative DNA lesions generated in organelle genomes due to reactive oxygen species (ROS) (27). Long-patch BER is reported in *P. falciparum* (28,29). Candidate *P. falciparum* apicoplast/mitochondrial DNA glycosylases/lyases that recognize and remove oxidised bases to generate apurinic/apyrimidinic (AP) sites have been identified but their organellar targeting is not yet confirmed (30,31). Interestingly, the two parasite nuclear-encoded Class II AP endonucleases that hydrolyze abasic sites in BER operate exclusively in the mitochondrion (30,32). Short-patch BER requires DNA polymerase-mediated removal of the 5′-deoxyribose phosphate (5′-dRP) generated after AP-site cleavage. A deficit of dRP lyase activity causes a switch to long-patch BER where the displaced strand (flap) carrying the 5′-dRP is cleaved by a Flap endonuclease (FEN). Flap endonucleases act as both structure-specific endonucleases and 5′-3′ exonucleases to process DNA structures generated during DNA replication, repair, and recombination (33,34, reviewed in (35)).

5′-3′ Exonuclease/FEN activity either resides in independent protein units or in a domain fused to bacterial/bacteriophage DNA polymerase I. Among the former, eukaryotic EXO1 which has 5′-3′ exonuclease, 5′-flap cleavage and 5′-3′ RNaseH activities participates in mismatch repair (MMR) to remove the mismatch-containing DNA strand from the 5′-3′ direction starting from a pre-introduced nick/gap (36,37) (Supplementary Table S1). It also removes 5′-primers from nascent Okazaki fragments, can cleave short (1–2nt) 5′-flaps generated by strand displacement, functions in DNA end resection for replication fork remodelling/restart of stalled forks, and generates 3′-ssDNA overhangs for DNA recombination events in double-strand break repair (DSBR) (38, reviewed in (39) (Supplementary Table S1). Among the exonuclease/FEN fused with DNA polymerases, the N-terminal 5′-3′ exonuclease/FEN domain within the multidomain (encompassing distinct polymerisation, 3′-5′ proofreading and 5′-3′ exonuclease functions) bacterial DNA polymerase I family allows the protein to degrade RNA primers on the lagging strand and act as a structure-specific 5′-endonuclease to remove DNA strand ahead of the site of polymerase addition (40). The *E. coli* DNA polymerase I homolog from *Thermus aquaticus* (Taq pol) has an N-terminal 5′-3′ exonuclease domain and C-terminal polymerase domains but its intervening vestigial 3′-5′ exonuclease domain has lost 3′-5′ editing activity (41,42). The DNA polymerase domain of apicoplast *Pf*Prex is phylogenetically closest to DNA polymerase I of the thermophilic bacterium *Aquifex aeolicus* (43); it contains 3′-5′ proofreading activity (19) but lacks a 5′-3′ exonuclease/FEN domain.

We addressed the question of how 5′-flap cleavage in strand-displacement synthesis during Okazaki fragment maturation at DNA replication forks or in DNA repair is catalysed in the apicoplast. We selected a putative 5′-3′ exonuclease (*Pf*Exo) with sequence motif conservation with *Mycobacterium smegmatis* FenA and the 5′-3′ exonuclease/resolvase domain of Taq polymerase. The protein carried a long LCR insertion conserved only in primate-infecting *Plasmodium* species belonging to subgenus *Laverania*. The apicoplast-targeted recombinant *Pf*Exo expectedly functioned as a 5′-3′ exonuclease/FEN on dsDNA, but had additional bipolar exonuclease activity on ssDNA and RNA–DNA hybrids. The absence of a human *Pf*Exo homolog, its likely essentiality for parasite survival in blood stages, and interaction of *Pf*Exo with *Pf*Prex identified the unique *Plasmodium* protein as a contributor to DNA transactions in the apicoplast. The limited substrate range of the *P. berghei* ortholog lacking the LCR also provided evidence for the role of a *P. falciparum* LCR in enzymatic function.

## Materials and methods

### Sequence analysis and phylogeny

Putative homologs of *Pf*Exo (*PF*3D7_0203900, PlasmoDB) were identified by BlastP. Multiple sequence alignments were performed by ClustalW. Signal and transit peptide predictions were done by SignalP 5.0 and PlasmoAP (44) tools, respectively. Disordered regions of *Pf*Exo were predicted by Disopred (45) analysis.

A phylogenetic tree containing 69 putative 5′-3′ exonuclease domain was constructed using automated MEGA X (46). Sequences of the orthologs were obtained from OrthoMCL (47), and also identified using BlastP and UniProt. Protein sequences were aligned using MUSCLE and evolutionary history was inferred using the Maximum Likelihood method and Whelan Goldman Model (48). Initial tree for the heuristic search was constructed automatically by applying Neighbour-Join and BioNJ algorithms to a matrix of pairwise distances estimated using the JTT model, and then selecting the topology with superior log likelihood value. All positions with <95% site coverage were eliminated (partial deletion option). A minimum of 1000 bootstrap replicates were analysed.

### Molecular structure modelling

*Pf*Exo was modelled using automated Swiss-Model (49). The crystal structure of *M. smegmatis* FENA (PDB: 6C33) (50) was used as template for *Pf*Exo. The models were evaluated by secondary structural analysis using Ramachandran plots generated by Procheck (51) and the RMSD scores were obtained by aligning the proteins to the template using PyMol (The PyMOL Molecular Graphics System, Version 1.2r3pre, Schrödinger, LLC).

### Recombinant protein expression and purification

The gene segment encoding *Pf*Exo (91–577 amino acids) was PCR-amplified using specific primers (Supplementary Table S2) with *P. falciparum* 3D7 genomic DNA as template. *Pb*Exo (*PB*ANKA_0301700, 115–453 amino acids) was amplified from *P. berghei* ANKA DNA as template using gene specific primers (Supplementary Table S2). The PCR-amplified segments were cloned into BamH1 and Sal1 (*Pf*Exo) and BamH1 and HindIII (*Pb*Exo) sites in the pET-23a vector carrying 6X-His tag at the C-terminal, and confirmed by DNA sequencing. The recombinant proteins were expressed in *Pf*Exo-pET23a- or *Pb*Exo-pET23a-transformed *E. coli* BL21-DE3 Codon Plus and grown in LB media supplemented with ampicillin (100 μg/ml) and chloramphenicol (25 μg/ml) till OD_600_ reached 0.6, followed by induction with 0.5 M IPTG at 20°C for 16 h. The cells were harvested by pelleting at 3663 × g for 10 min at 4°C and resuspended in Buffer A [50 mM Tris–HCl pH 8.0, 300 mM NaCl, 5% glycerol, 0.3% N-lauryl sarcosine, 1 mM PMSF and 1× protease inhibitor cocktail (Sigma Aldrich, USA, #S8830)] for *Pf*Exo and Buffer B (50 mM Tris–HCl pH 8.0, 300 mM NaCl, 5% glycerol, 1 mM PMSF and 1× protease inhibitor cocktail) for *Pb*Exo. After sonication, the homogenates were centrifuged at 10 174 × g for 45 min at 4°C and the clarified cell lysates were passed through a pre-equilibrated Ni-nitrilotriacetic acid (Ni-NTA) Superflow column (Qiagen, Germany, #30410). After washing, the proteins were eluted using 300 mM imidazole in Buffer B (without N-lauryl sarcosine). The eluates were pooled and concentrated. *Pb*Exo and *Pf*Exo were further purified by size exclusion chromatography (SEC) using a Superdex S-75 column (GE Healthcare, USA) in an AKTA purifier system (GE Healthcare) in a refrigerated cabinet. The chromatography buffer contained 50 mM Tris–HCl pH8.0, 50 mM NaCl and 5% glycerol. The peak fractions were checked by SDS-PAGE and those containing pure protein were pooled and concentrated using Centricon filters (Merck, USA) and checked by 10% SDS-PAGE. The final concentration of the proteins was determined by bicinchoninic acid (BCA; Thermo Fisher Scientific, USA, #23227) assay.

DNA encoding *Pf*Prex Exo/Pol domain (residues 1360–2016) was PCR-amplified (primers in Supplementary Table S2) and cloned at the BamH1–Sal1 sites in pET23a. The protein was expressed in *E. coli* BL21-DE3 Codon Plus and purified identically to *Pf*Exo.

For expression of N-terminal glutathione S-transferase (GST)-tagged *Pf*Exo (GST-*Pf*Exo), the *Pf*Exo gene segment (91–577 amino acids) was cloned as a BamHI-SalI insert in pGEX-KG. The fusion protein was expressed in *E. coli* BL21 (DE3) in the presence of the RIG plasmid. The cells were harvested as described above and resuspended in Buffer B. GST-*Pf*Exo was purified through Protino glutathione agarose 4B beads (Machery Nagel, Germany, #745500.10) with elution using 50 mM reduced glutathione (Amresco, USA, #0399). The eluted protein was concentrated in Centricon filters with buffer exchange (50 mM Tris–HCl pH8.0, 50 mM NaCl and 5% glycerol) and checked on SDS-PAGE.

### Deletion/insertion and site-directed mutagenesis

The *Pf*Exo insertion region (amino acids 241–393) was deleted by overlapping deletion mutagenesis. Fragments of 450 and 552 bp flanking the region to be deleted were PCR-amplified using forward and reverse primers and internal primers that harboured sequences flanking the insertion (Supplementary Table S2), with *Pf*Exo-pET23a as template. The two fragments thus obtained were annealed and extended (Supplementary Table S2). The resulting DNA was then amplified and cloned into pET23a at BamH1 and Sal1 sites and confirmed by DNA sequencing. *Pf*ExoΔins was expressed as a recombinant protein in *E. coli* BL21-DE3 CodonPlus cells. Post harvesting, the cells were resuspended in Buffer C (50 mM Tris–HCl pH 7.5, 300 mM NaCl, 5% glycerol, 1 mM PMSF) and sonicated. The homogenized lysate was centrifuged at 10 174 × g for 30 min at 4°C to separate the soluble supernatant and insoluble pellet fractions. The pellet was washed twice with Buffer C and centrifuged at 10 174 × g at 4°C for 30 min each, followed by two washes with 0.05% Triton X-100 in Buffer C. The detergent was removed by two washes with Buffer C. The pellet was resuspended in Buffer D (50 mM Tris–HCl pH 7.5, 300 mM NaCl, 5% glycerol, 1 mM PMSF, 0.2% N-lauryl sarcosine) and sonicated at 28% amplitude for 40 min (20 s ON/20 s OFF cycle). The homogenate was centrifuged at 10 174 × g for 45 min at 4°C and the supernatant was passed through a pre-equilibrated Ni-NTA column. The protein was eluted by 300 mM imidazole in Buffer C. The eluate was concentrated, dialyzed in Buffer E (50 mM Tris–HCl pH7.5, 50 mM NaCl, 5% glycerol) and checked on 10% SDS-PAGE.

The mutants for *Pf*Exo (*Pf*ExoC201A, *Pf*ExoC392A, *Pf*ExoH393A, *Pf*ExoC436A, *Pf*ExoC488A, *Pf*ExoC532A, *Pf*ExoC436A-C532A, *Pf*ExoD218N, *Pf*ExoD217A, *Pf*ExoD417A, *Pf*ExoD417A-470A-473A and *Pf*ExoD217A-470A-473A) were generated by NEB Q5 site-directed mutagenesis kit, using *Pf*Exo-pET23a as template. Mutations were confirmed by DNA sequencing. The recombinant mutant proteins were purified as described for *Pf*Exo.

Residues 241–393 of *Pf*Exo comprising its insertion sequence were incorporated into *Pb*Exo between residues 154 and 155 by gene synthesis (GenScript Biotech, Singapore) and cloning in pET23a. The recombinant protein (*Pb*Exo-ins+) was expressed and purified as for *Pb*Exo.

### Parasite culture

*P. falciparum* 3D7 parasites (source: MR4, BEI Resources, ATCC, USA) were cultured in human RBCs (approval #CDRI/IEC/2017/A4 by Institutional Ethics Committee-Human Research) at 2% haematocrit in RPMI-1640 medium (HEPES-modified, Sigma Aldrich, USA, #23400021) supplemented with 0.2% sodium bicarbonate, 1% glucose, 100 μM hypoxanthine (Sigma Aldrich, USA, #H9377), gentamycin (25 μg/ml) and 0.5% Albumax II (Invitrogen, USA, #11021045). Parasite extract was obtained after releasing parasites from RBCs by 0.5% saponin lysis, washing with phosphate buffered saline (PBS), and suspension in 5× SDS loading buffer.

### Antibody generation and western blotting

Purified recombinant *Pf*Exo was used to raise antisera by subcutaneous immunization in rabbit (New Zealand White). Animal use approval was obtained from the Institutional Animal Ethics Committee (#IAEC/2007126/(292-5/18) Ren-11). 200 μg of protein emulsified in Freund's complete adjuvant (CFA, Sigma Aldrich, USA, #F5881) was injected for primary immunization, followed by two doses of 100 μg protein in Freund's incomplete adjuvant (FA, Sigma Aldrich, USA, #F5506) as first and second booster given at an interval of 21 days. The rabbit was bled after 10 days of the second booster. Antibodies against *Pf*Prex Exo/Pol were raised in mice; primary immunization was with 50 μg protein (in CFA) /mouse followed by two booster doses of 25 μg protein (in FA)/mouse after a gap of 10 days each. The antisera were checked against recombinant proteins and were used to detect the protein in parasite lysate [antiserum dilution: 1:100 (*Pf*Exo) and 1:250 (*Pf*Prex Exo/Pol) as primary Ab] with HRP-conjugated goat anti-rabbit IgG (1:10000; Santa Cruz Biotechnology, USA, #AP187P) or HRP-conjugated goat anti-mouse IgG (1:5000; Sigma-Aldrich, USA, #A0168) as secondary antibody. The blots were developed by chemiluminescence using ECL kit (Merck Millipore, USA, #WBKLS0500). Anti-*Pf*Exo Ab was purified against the antigen by nitrocellulose immobilization (52).

Recombinant proteins were detected in western blots using anti-6XHis Ab (1:1000; Santa Cruz Biotechnology, USA, #SC-53073) as primary antibody and HRP-conjugated goat anti-mouse (1:5000; Sigma Aldrich, USA, #A0168) as secondary antibody. The blots were developed using the ECL kit.

### Immunofluorescence assay and confocal microscopy

*P. falciparum*-infected RBCs primarily at the trophozoite stage and parasitemia of 10–15% were processed for immunofluorescence labelling and confocal microscopy as described by (13). Cells were fixed in PBS containing 4% *para*-formaldehyde and 0.0075% (v/v) glutaraldehyde for 30 min. Fixed cells were washed with PBS twice before permeabilization with 0.1% (v/v) Triton X-100 in PBS for 15 min at room temperature. After multiple washes with PBS, cells were blocked in 3% BSA in PBS for 1 h at 4°C and incubated overnight in purified anti-*Pf*Exo Ab (1:50) and anti-*Pf*HUp Ab (1:100) (13), the latter used as a marker for the apicoplast. After washing five times with PBS, cells were probed with Alexa Fluor 568-tagged anti-rabbit Ab (Invitrogen, USA, #A11011) and Alexa Fluor 488-tagged anti-mouse Ab (1:1000) (Invitrogen, USA, #A11001) in 3% BSA. DAPI (20 μg/ml) prepared in PBS was added to the secondary Ab mix. Cells in the secondary Ab mix were layered on poly L-lysine coated glass cover slips for 2 h at room temperature. For mitochondrial staining, cells were incubated with 50 nM Mitotracker Red CMXROS (Invitrogen, USA, #M7512) for 30 min at 37°C prior to fixing; anti-rabbit Alexa Fluor 514-tagged Ab was used as secondary Ab for *Pf*Exo in Mitotracker-stained cells. After incubation, coverslips were washed five times with 500 μl cold PBS gently to remove un-adhered cells. Cover slips were mounted in anti-fade mounting media and imaging was carried out on a Leica SP8 confocal microscope using 63× oil-immersion objective.

### *In vitro* DNA cleavage assays

The DNA/RNA substrates used for the exonuclease and flap cleavage activities (Supplementary Table S3) were synthesized by Integrated DNA Technologies, USA.

- 5′-3′ exonuclease assay: 5′-3′ exonuclease activity was checked on both dsDNA and ssDNA substrates. The dsDNA substrates included 3′ FAM-labelled 5′- recessed, blunt-end and 1-nt gapped substrate while the ssDNA substrate was a 3′ FAM-labelled 40 bp oligonucleotide. The 20 μl reaction contained 100 nM DNA substrate, 50 pmol protein, 50 mM Tris–HCl pH 8.5, 50 mM NaCl, 2 mM MgCl_2_ and 100 μg/ml BSA (41,50). The reactions at 37°C were stopped at different time points post incubation by addition of 10 μl stop solution (98% formamide, 10 mM EDTA and 0.015% Orange-G). Samples were heat-denatured at 100°C for 10 min and snap-chilled in ice. The reaction products were separated on 8 M urea–20% PAGE in Tris-borate (90 mM)-EDTA (2 mM) buffer (pH 8.3) and visualized using Cy2/Cy3 filter in an Image Quant Las 4000 Chemidocumentation system (Cytiva, USA). 6-FAM labelled oligonucleotides were used as markers. N^6^-6 Aminohexyl-ATP-6FAM (molecular weight: 964.6 g/mol) was the lowest marker that would migrate below the dinucleotide 5′-AA/6FAM/-3′ (mw: 1212.96 g/mol) and just above the mononucleotide 5′-A/6FAM/-3′ (mw: 900.66 g/mol).
- 3′-5′ exonuclease assay: The 3′-5′ exonuclease activities were checked on 5′ FAM-labelled recessed dsDNA and ssDNA substrates. The reaction conditions were the same as described for 5′-3′ exonuclease activity.
- Flap endonuclease assay: The structure-specific endonuclease activity was checked on both 5′-flap and 3′-flap substrates at the same reaction conditions as the 5′-3′ exonuclease assay.
- RNase assay: The RNase activity of the proteins was checked on 3′ and 5′ FAM-labelled RNA–DNA hybrid substrates. To avoid RNase contamination, the tubes, tips and buffer solutions used for the assays were DEPC-treated. The 20 μl reaction mixtures containing 100 nM substrate, 50 pmol protein, 50 mM Tris–HCl pH 8.5, 50 mM NaCl, 2 mM MgCl_2_ and 100 μg/ml BSA were incubated at 37°C and the reactions were stopped at different time points. *E. coli* RNaseH (New England Biolabs, USA, #M0297) was used as positive control and identically purified *Pf*KsgA1 served as negative control. The products were separated on 8 M urea-20% PAGE.
- pH and temperature dependence: pH-dependence of exonuclease activity was assayed in buffers containing 50 mM HEPES-KOH (pH 6–7) with 50 mM KCl, or 50 mM Tris–HCl (pH 7.5–9.5) with 50 mM NaCl. Temperature dependence was checked by subjecting the reaction mixtures to 30 min incubation at 37, 50, 75 and 100°C. The reactions were stopped and the products were separated on 8 M urea–20% PAGE.
- Percent exonuclease activity: Percent exonuclease on dsDNA and ssDNA was determined in assays using 0.25–50 pmol protein. Band intensities were measured by Image Quant TL (Cytiva, USA). Percent exonuclease activity was calculated by the equation below (53):
  \begin{equation*}{\mathrm{\%\, exonuclease\, activity}}\,{\mathrm{ = }}\frac{{{\mathrm{[}}\left( {{\mathrm{N - 1}}} \right){\mathrm{ \pm 2}}\left( {{\mathrm{N - 2}}} \right){\mathrm{ \pm 3}}\left( {{\mathrm{N - 3}}} \right){\mathrm{ \ldots ]}}}}{{{\mathrm{N + }}\left[ {\left( {{\mathrm{N - 1}}} \right){\mathrm{ + 2}}\left( {{\mathrm{N - 2}}} \right){\mathrm{ + 3}}\left( {{\mathrm{N - 3}}} \right){\mathrm{ \ldots }}} \right]}}\end{equation*}
  where *N* is the amount of the intact 40-mer substrate, *N*– 1, *N*– 2, *N*– 3…are amounts of the subsequent exonuclease products.

### Statistical analysis

The percent exonuclease activity of *Pf*Exo and its mutants were calculated as mean and standard deviation of three separate titration experiments and plotted as function of pmoles of protein used. The datasets for each variant were subjected to linear regression analysis in GraphPad Prism 5, and the slopes of the titration curves thus obtained were compared to derive the specific activities of each mutant post normalization to that of *Pf*Exo.

### DNA binding

The binding of the proteins to various DNA substrates was analysed by electrophoretic mobility shift assay (EMSA). 400 nM FAM-lablelled DNA was incubated with varying concentrations of the proteins in the presence of binding buffer (200 mM HEPES pH 8.0, 500 mM NaCl, 10 mM DTT, 1mg/ml BSA,10% glycerol and 2 mM MgCl_2;_ final reaction volume of 20 μl) for 30 min in ice followed by addition of the loading dye (50% glycerol, 1× Tris–borate–EDTA buffer and 0.015% bromophenol blue). The reaction products were separated on 6% native PAGE.

### Biolayer interferometry (BLI) based DNA binding analysis

The binding affinity of *Pf*Exo and *Pf*ExoΔins on blunt-end dsDNA was measured by BLI experiments designed using the eight channels in OctetR8 instrument (Sartorius, Germany). 300 nM biotin-labelled blunt-end DNA (34-mer) was immobilized on pre-hydrated Streptavidin biosensors (Sartorius, Germany, #18-5019) to reach a loading of 0.6 nm. Following the loading cycle, the DNA-coated biosensors were dipped into varying concentrations of the analytes (*Pf*Exo and *Pf*ExoΔins) and subjected to association for 200 s followed by dissociation in the reaction buffer (1× kinetic buffer diluted in 1× PBS) for 300 s. The binding shifts thus recorded were plotted and the respective KD values were analysed using the Octet Analysis Studio software 12.2.2.26. Prior to fitting, all the datasets were reference subtracted, aligned to Y axis and interstep correction was carried out. The sensograms for varying concentrations of analytes for each protein were fit globally using 1:1 binding model and the KD value thus obtained from the kinetic analysis was recorded. The first 200 s of association and 120 s of dissociation were taken into account at the time of fitting. The experiments were done in replicates to ensure reproducibility.

### Metal content quantification

Plasticware and glassware used for metal assays was washed with Chelex 100 (Biorad Laboratories, USA, #143-2382) treated water. Manganese quantification assay was based on the colorimetric method as described by (54). The purified protein was subjected to overnight chelation by 5 mM 1,10-phenanthroline (VWR Life Science, USA, #0516) to remove bound metal ions, followed by dialysis in 50 mM Tris–Cl pH 8.0 and 50 mM NaCl to remove the chelator. The dialyzed protein was incubated with 5 mM MnCl_2_ for 45 min, after which the reaction mixture was dialyzed again in metal-free buffer. The protein was precipitated with 2.1% (w/v) perchloric acid and the supernatant was neutralized with 8.2 M KOH. After chilling and removal of KClO_4_ crystals, 50 μl of the deproteinized supernatant was mixed with 200 μl assay buffer [43.1 mM K_3_PO_4_ pH 7.8, 384 μM *ortho*-dianisidine, 0.024% (w/v) Triton X-100, 10.9 μM riboflavin and 96 μM bovine liver catalase (Sigma Aldrich, USA, #C9322)] and exposed to two full visible spectral range bulbs for 8 min. Absorbance of the coloured complex thus formed was measured at 460 nm and quantification was done based on the standard curve obtained using manganese standard solution (Sigma Aldrich, USA, #77036) for atomic absorption spectroscopy.

### Spectral scanning for [Fe–S] and conversion to apo-form

The proteins were subjected to spectral scanning in the UV–visible range (270–800 nm) to check for the presence of [4Fe–4S] cluster. For conversion of *Pf*Exo to its apo-form, the [4Fe–4S] containing holo-protein purified in an anaerobic chamber was incubated with EDTA and potassium ferricyanide in a molar ratio 1:50:20 (protein: EDTA: K_4_FeCN_6_) for 10–15 min after which the reaction mixture was passed through a pre-equilibrated NAP5 column (GE Healthcare, USA, #17-0853-02) (55). The protein was finally eluted in 50 mM Tris–HCl pH 8.0 and 50 mM NaCl.

### Protein pulldown assay

Parasite were harvested with 0.05% saponin lysis and washed twice with chilled 1× PBS. The pellet was lysed using lysis buffer [50 mM Tris–HCl pH 7.5, 300 mM NaCl, 1% Triton X-100, 2 mM EDTA and 1× protease inhibitor cocktail (Sigma Aldrich, USA, #P8340)] for 30 min at 4°C. The lysate was centrifuged at 4°C at 13 800 × g for 20 min to remove cell debris and precleared using Ni-NTA beads for 1 h at 4°C. The precleared supernatant obtained after centrifugation at 13 800 × g for 10 min was then incubated with 25 μg of recombinant 6X-His tagged *Pf*Prex Exo/Pol or *Pf*Exo overnight at 4°C. 30 μl 50% Ni-NTA slurry was added to the reaction mixture and incubated for 2 h at 4°C followed by pelleting and subsequent washing of the beads with chilled 1× PBS. Protein was eluted using 300 mM imidazole after incubation at 4°C for 15 min. Samples were made in 1× Laemlli buffer, boiled at 100°C for 5 min, and separated on SDS-PAGE followed by probing with anti-*Pf*Exo sera (1:100), anti-*Pf*Prex Exo/Pol sera (1:250), anti-*Pf*YihA sera (1:250), anti-*Pf*HUp sera (1:4000) or anti-6XHis Ab. Beads alone and *E. coli* EngA as bait were used as controls.

Interaction of recombinant *Pf*Exo and *Pf*Prex Exo/Pol was assayed by using GST-*Pf*Exo bound to glutathione agarose beads as bait. GST alone was used as negative control bait. 30 μg of bait protein (purified GST-*Pf*Exo or GST) was bound to 100 μl of Protino Glutathione Agarose 4B pre-equilibrated with binding buffer (50 mM Tris–HCl pH 8.0, 150 mM NaCl, 1 mM EDTA)] by incubation for 2 h at 4°C on a tube rotator. Beads were washed once with wash buffer (50 mM Tris–HCl pH 8.0, 150 mM NaCl, 0.1% Triton X-100, 1 mM EDTA) and twice with binding buffer. 30 μg purified *Pf*Prex Exo/Pol (with C-terminal 6XHis tag) was added to GST-*Pf*Exo- or GST-bound beads in 1 ml binding buffer. The mix was incubated overnight at 4°C on tube rotator. The beads were pelleted and the supernatant collected as flow through. The beads were then washed two times with wash buffer followed by elution with 50 mM reduced glutathione in 200 μl wash buffer. Samples were separated on SDS-PAGE followed by western blotting with anti-6XHis antibody and anti-GST antibody (Santa Cruz Biotechnology, USA, #sc-138 HRP).

### Gene deletion of *Pb*Exo

The attempts to disrupt *PbExo* (*PB*ANKA_0301700) were performed with a replacement plasmid containing GFP reporter and hDHFR:yFCU selection marker (56). Two 5′- and 3′-UTR homology regions F1 and F2 (0.57 Kb) were amplified using primers 1430/1431 and 1432/1433 and cloned at SalI and NotI/AscI respectively in the *Pb*C-GFP-hDHFR:yFCU vector. The construct was linearized with XhoI/AscI and transfected into *P. berghei* (MRA-311, BEI resources, USA) schizonts as previously described (57). Transfected parasites were selected by treatment of the mice with pyrimethamine. The transfection efficiency was calculated as described previously (57). Genomic DNA was isolated from resistant parasites and diagnostic PCR was performed using specific primer sets 1538/1215 and 1539/1225 for 5′ and 3′ integrations respectively. Primers are listed in Supplementary Table S4. All animal experiments performed in this study were approved by the Institutional Animal Ethics Committee at CSIR-Central Drug Research Institute, India (approval numbers: IAEC/2017/265b and IAEC/2022/4).

## Results

### Identification of an exonuclease with possible role in organellar DNA replication/repair

Mining the *P. falciparum* nuclear genome for putative 5′-3′ exonucleases/FENs identified a FEN homolog with predicted mitochondrial targeting (30) and two homologs (*PF*3D7_0203900 and *PF*3D7_0204600) carrying the bipartite apicoplast targeting element. Genome wide insertion mutagenesis has indicated that *PF*3D7_0203900 is essential (58); knockout of its *P. berghei* ortholog (*PB*ANKA_0301700) results in a significantly slow growth phenotype (59,60). Since *PF*3D7_0204600 seems to be dispensable in *P. falciparum* (58), we prioritised *PF*3D7_0203900 (henceforth called *Pf*Exo) for functional characterization.

*Pf*Exo exhibits sequence similarity with the 5′-3′ exonuclease/N-ter resolvase-like domain of Taq DNA polymerase (27.7% identity) and the characterized FenA of *M. smegmatis* (21.84% identity) (42,50). Multiple sequence alignment shows that *Pf*Exo has conserved Asp residues as in the *Ms*FenA active site, but is distinguished by the presence of a large 175 aa LCR insertion (residues 223–397) not found in homologs from other organisms (Supplementary Figure S1). Phylogenetic analysis of *Pf*Exo homologs (Supplementary Figure S2) showed that apicomplexan and chromerid orthologs (Alveolates) formed a clade which was distinct from the Euglenozoa, Oomycota, Viridiplantae and bacterial clusters. Among apicomplexan parasites, a *Pf*Exo ortholog seems to be absent in *Cryptosporidium* which lacks the apicoplast and has a reduced mitochondrion (mitosome) (61). *Pf*Exo is closest to orthologs from other primate-infecting *Plasmodium* species of the subgenus *Laverania* which contain variable lengths of the insertion sequence absent in all other *Plasmodium* species (Supplementary Figure S2, Supplementary Figure S3A, B). The insertion in *Pf*Exo is predicted to be moderately disordered and contains stretches rich in Asp residues contributing to a net negative charge in the region (Supplementary Figure S3C, D).

**Figure 1.**
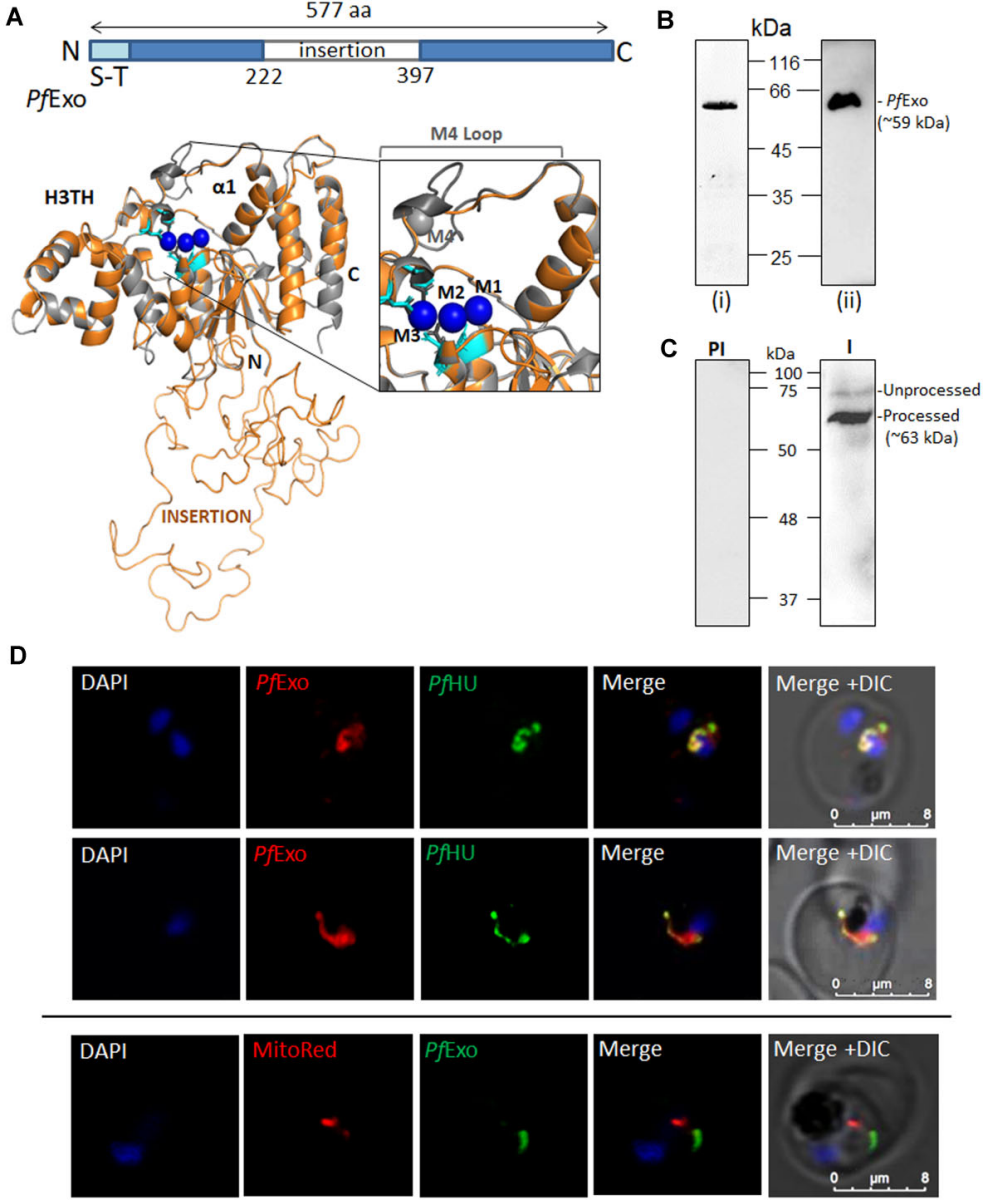
Molecular structure modelling of *Pf*Exo and its subcellular localization. (**A**) *Pf*Exo (orange) was modelled on the crystal structure of *M. smegmatis* FenA (grey). The H3TH domain and α1 helix are marked. The large insertion (LCR) in *Pf*Exo could not be modelled and appears as a loop. The magnified active site shows conserved active site residues in *Pf*Exo (D217, D417, D470, D473) (cyan) at the M1, M2, M3 metal–binding sites and the region corresponding to the M4 loop of *M. smegmatis* FenA which is further shortened in *Pf*Exo. (**B**) Coomassie-stained SDS-PA gel of purified recombinant *Pf*Exo (i), and western blot of the purified protein using anti-6XHis Ab (ii). (**C**) Anti-*Pf*Exo Abs recognized a specific ∼63 kDa band and a faint ∼68 kDa band in *P. falciparum* lysate possibly representing processed and unprocessed bands of the protein, respectively. PI, pre-immune serum; I, immune serum. (**D**) Immunofluorescence confocal microscopy of erythrocytic *P. falciparum* trophozoites localized *Pf*Exo with the apicoplast marker *Pf*HU. The top two rows show scans of different infected erythrocytes using anti-*Pf*Exo and anti-*Pf*HU Abs. There was no overlap of *Pf*Exo signal with Mitotracker Red (bottom row). DAPI was used as nuclear stain. The experiment was repeated three times; images shown are representative of at least 30 images scanned for each set.

Homology structure modelling of *Pf*Exo on *Ms*FenA (RMSD = 0.193 Å) (Figure 1A) revealed conservation of structural domains including the acidic residues which bind the three active site metal ions (M1, M2, M3). The large disordered *Pf*Exo insertion could not be modelled and appeared as a loop. The helix-turn-helix (H3TH) motif of other FEN and FEN-like enzymes which engages the segment of duplex DNA that immediately precedes the duplex-flap junction (50,62) and the wedge helix (corresponding to α1 of *Ms*FenA) that bends the DNA at the flap/nick junction (50,62) before cleavage of the scissile phophodiester bond by metal-catalysed hydrolysis are also conserved in *Pf*Exo. FENs and FEN-like family proteins such as bacteriophage T5 FEN, human FEN 1, human EXO1 and T4 RNaseH contain a helical arch (62–65) that forms an aperture through which the 5′ flap threads. This helical arch which also binds the fourth active site metal ion (M4) is replaced by a loop in both *Ms*FenA and *Pf*Exo, the *Pf*Exo loop being shorter of the two (Figure 1A; Supplementary Figure S1) (50). All active site residues at M1, M2 and M3 are conserved between *Ms*FenA and *Pf*Exo. These comprise *Pf*Exo M1 site residues Asp114, Asp164, Glu215 and Asp217 (corresponding to Asp9, Asp60, Glu123 and Asp125 of *Ms*FenA), M2 site residues Asp217, Asp415, Asp417, Asp473 (corresponding to Asp125, Asp146, Asp148 and Asp208 of *Ms*FenA), and M3 site residues Asp417, Asp470, Asp473 (corresponding to Asp148, Asp205, Asp208 of *Ms*FenA). *Ms*FenA contains a fourth metal ion (M4) engaged through metal contacts and a salt bridge (Arg64-Asp90) (50); none of the side chain ligands involved in the interactions at the *Ms*FenA M4 loop are conserved in *Pf*Exo suggesting that it might differ from *Ms*FenA in having only three metal sites.

### *Pf*Exo is targeted to the apicoplast

In order to functionally characterize *Pf*Exo, the conserved region of the protein lacking the N-terminal extension which contains the predicted weak signal sequence and transit peptide was recombinantly expressed. The N-terminal end of recombinant *Pf*Exo was at residue 91, immediately after a poly-lysine stretch, and was based on sequence conservation with *Ms*FenA and 5′-3′ exonuclease/N-terminal resolvase-like domain of Taq DNA polymerase (Supplementary Figure S1). The C-terminal 6X-His-tagged fusion protein of ∼59 kDa was purified by affinity chromatography followed by SEC (Supplementary Figure S4A) and yielded *Pf*Exo of >95% purity as checked by SDS-PAGE (Figure 1B). Anti-*Pf*Exo antibodies generated against the purified protein in rabbit detected a specific major band of ∼63 kDa and a band of ∼68 kDa in the parasite lysate, the latter corresponding to the expected size of the unprocessed protein (Figure 1C). The size of the major band suggests N-terminal processing of *Pf*Exo between amino acid 50–55 (Supplementary Figure S1). The ∼35 residues from the N-terminal processing site that have been excluded in recombinant *Pf*Exo are not present in its closest homologs (*Ms*FenA and resolvase domain of Taq polymerase) and are not conserved in the long N-terminal extension of its *Arabidopsis thaliana* homolog (Supplementary Figure S1), suggesting that they are unlikely to be functionally relevant.

Immunofluorescence localization of the protein in *P. falciparum* infected erythrocytes using purified anti-*Pf*Exo antibodies, co-localized *Pf*Exo signals with the apicoplast marker *Pf*HU (Figure 1D). No overlap was seen with the mitochondrial marker dye or with DAPI-stained nuclei, indicating that *Pf*Exo is targeted to the parasite apicoplast and does not function in the mitochondrion or nucleus.

### *Pf*Exo has a broad DNA substrate range and exhibits bipolarity on ssDNA and RNA–DNA hybrids

**Figure 2.**
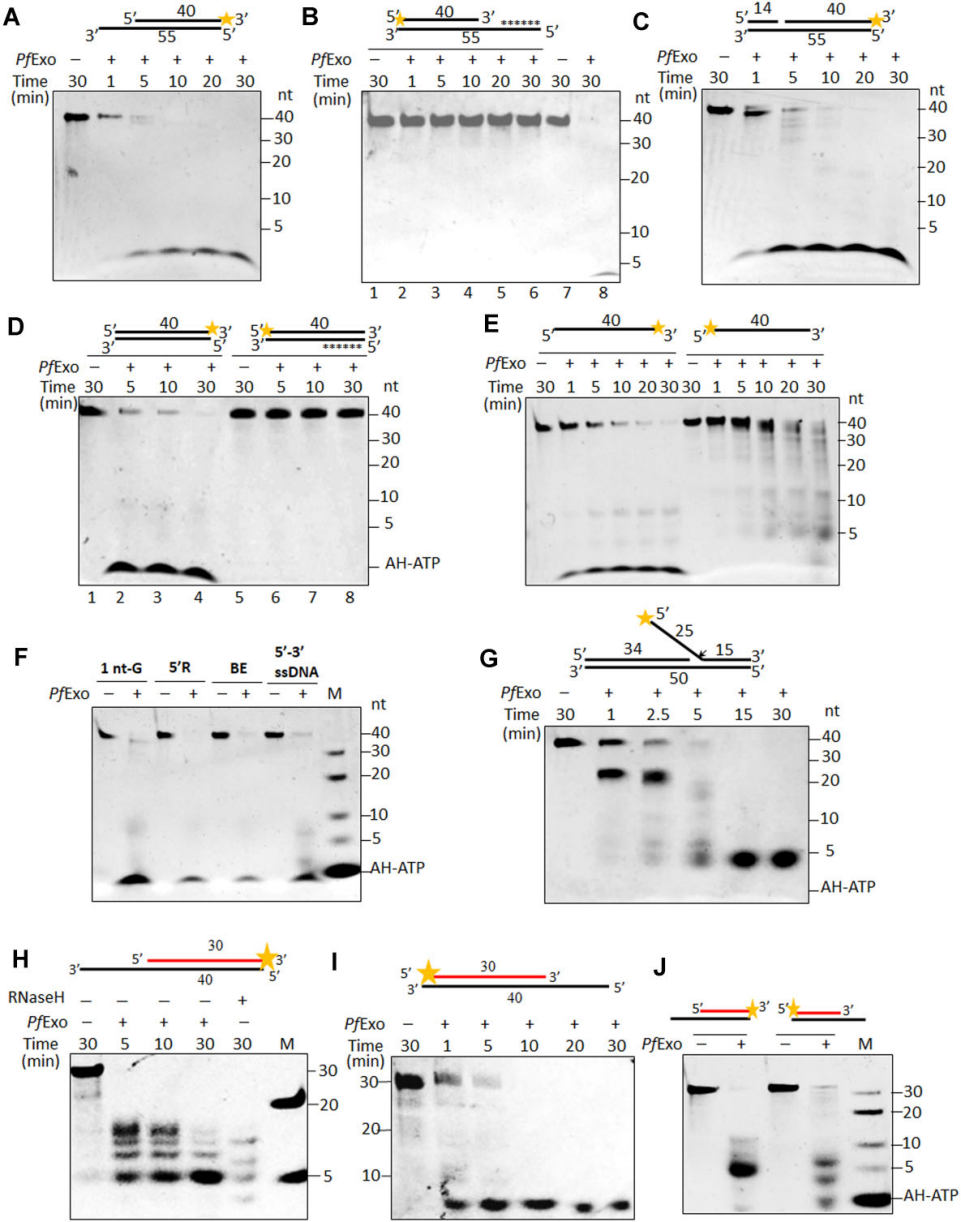
Exo-/endo-nucleolytic activity of *Pf*Exo on nucleic acid substrates. Time-dependent exonuclease activity on 5′-recessed dsDNA (**A**), 3′-recessed dsDNA (**B**), 1 nt-gapped (**C**) and blunt-end dsDNA (**D**) substrates. Cleavage of 5′-recessed DNA by *Pf*Exo (lanes 7–8) served as positive control for enzyme activity in (B). Asterisks in (B) and (D) denote six consecutive phosphorothioate bonds used for blocking the free 5′ end in order to detect possible 3′-5′ exonuclease activity on dsDNA substrates. (**E**) Assay of *Pf*Exo 5′-3′ and 3′-5′ exonuclease activity on ssDNA. (**F**) Terminal product size after cleavage of 1 nt-gapped (1nt-G), 5′-recessed (5′-R) and blunt-end (BE) dsDNA, and 5′-3′ ssDNA for 30 min. (**G**) 5′-flap removal by *Pf*Exo; arrow marks the endonuclease cleavage site that would generate the ∼25 nt flap cleavage product. RNase activity of *Pf*Exo on RNA–DNA hybrids in 5′-3′ (**H**) and 3′-5′ (**I**) polarity. (**J**) Terminal product size after 5′-3′ and 3′-5′ cleavage of RNA–DNA hybrids for 30 min. M, marker; AH-ATP, N^6^-6-Aminohexyl-ATP-6FAM. All experiments were repeated at least three times with different enzyme preparations; sizing experiments in (F) and (G) were repeated twice.

The 5′-3′ exonuclease activity of *Pf*Exo was first tested on 5′-recessed, 1 nt-gapped and blunt-end dsDNA substrates as well as on 3′-FAM labelled ssDNA (Figure 2A, C–E). The protein removed nucleotides from all substrates in a time-dependent manner. N^6^-6 Aminohexyl-ATP-6FAM was used as marker to determine the size of the lowest product; a terminal product of 1 nt was generated upon cleavage of dsDNA and ssDNA in 5′-3′ polarity (Figure 2F). Since rapid appearance of a terminal cleavage product without detectable intermediates was observed, we tested whether this was solely a result of cleavage of the dye-labelled mononucleotide from the 3′-end. ssDNA and dsDNA blocked by FAM-conjugated nucleotides at both termini, or 3′-FAM labeled ssDNA blocked by phosphorothioate linkages at the 5′-end, were resistant to cleavage by *Pf*Exo (Supplementary Figure S4B, C) thus ruling out this possibility and suggesting high processivity of the enzyme. In order to visualize intermediary exonuclease cleavage products by lowering the processivity of *Pf*Exo, we tested a range of suboptimal reaction conditions. A combination of higher salt (100 mM NaCl) and low temperature (20°C) allowed detection of intermediary cleavage products and their shortening over time (Supplementary Figure S4D–G). *Pf*Exo did not cleave 3′-recessed dsDNA (Figure 2B) or 5′-FAM labelled blunt-end dsDNA (Figure 2D); phosphorothioate bonds were incorporated in both substrates to block the free 5′-end. When tested for recognition and cleavage of DNA structures carrying 25 nt long 5′- or 3′-flaps, *Pf*Exo cleaved only the former (Figure 2G and Supplementary Figure S4H). A ∼25 nt flap cleavage product was generated in the 5′-flap cleavage reaction, followed by appearance of lower bands as the reaction progressed (Figure 2G). In order to check whether these bands represented endonucleolytic cleavage at multiple sites on the 25 nt 5′-flap or were products of exonucleolytic cleavage of the released flap, the 3′-5′ exonuclease activity of *Pf*Exo was tested on 5′-FAM labelled ssDNA. *Pf*Exo hydrolyzed ssDNA in the 3′-5′ direction and generated a major terminal product of 5 nt (Figure 2E). Thus the 5′-flap cleavage product could serve as substrate for further 3′-5′ exonucleolytic processing by the enzyme. Comparison of time-dependent exonuclease activity on 5′-3′ and 3′-5′ ssDNA showed that the terminal cleavage product appeared earlier with the former substrate, indicating better processivity of *Pf*Exo in the 5′-3′ direction (Figure 2E). The 3′-5′ exonuclease cleavage of ssDNA by *Pf*Exo was independent of DNA sequence composition (Supplementary Figure S4I). Hence, *Pf*Exo functions as a unidirectional (5′-3′) exonuclease on dsDNA but is bipolar on ssDNA.

We tested *Pf*Exo on ssDNA substrate lengths of 5–40 nt in both polarities. The enzyme was able to cleave the lowest length tested (5 nt) in the 5′-3′ direction but its cleavage efficiency on the 5 nt substrate was compromised compared to longer substrates (Supplementary Figure S5A). However, *Pf*Exo did not cleave the 5 nt substrate in the 3′-5′ direction as 5 nt is the terminal product size in this polarity (Supplementary Figure S5B).

pH dependence of exonuclease and flap endonuclease activities was assayed on 5′-recessed, 5′-FAM labelled ssDNA and 5′-flap substrates. *Pf*Exo could cleave the three substrates at a broad pH range (pH 6.0–8.5, 6.5–9.5 and 6–9, respectively) (Supplementary Figure S5C-E). It showed optimal 5′-3′ exonuclease activity at 37°C and 50°C with retention of some activity even at 75°C (Supplementary Figure S5F). Retention of *Pf*Exo activity at high temperature is a reflection of its homology to the N-ter resolvase domain of Taq pol. The protein functioned in the presence of both Mg^2+^ or Mn^2+^ ions (Supplementary Figure S5G), but not with Zn^2+^ or Ca^2+^ (data not shown). To measure the number of metal ions bound to *Pf*Exo, we removed metal ions by chelation and reconstituted the protein with Mn^2+^ alone. Each protein molecule was estimated to bind 2.77 ± 0.5 (mean ± SD) manganese atoms, in line with our structure model-based prediction of three metal sites on *Pf*Exo.

Considering its wide DNA substrate specificities and the fact that 5′-3′ exonucleases often have associated RNaseH activity, we tested *Pf*Exo on recessed 5′-3′ and 3′-5′ RNA–DNA hybrids. The protein could cleave both substrates with comparable efficiency (Figure 2H, I). *E. coli* RNaseH served as positive control (Figure 2H). To confirm that the observed RNase activity was not due to a contaminant in the purified *Pf*Exo preparation we set up negative control reactions with another identically purified parasite recombinant protein *Pf*KsgA1 (66) (Supplementary Figure S5H, I). The *Pf*Exo RNase major terminal product length was 5 nt in 5′-3′ polarity and 1 nt in 3′-5′ polarity (Figure 2J). Lowering *Pf*Exo processivity at suboptimal reaction conditions, as used for DNA substrates, enabled visualization of intermediary RNA cleavage products from RNA–DNA hybrids in both 5′-3′ and 3′-5′ polarities (Supplementary Figure S5J, K). As reported for *E. coli* RNaseH (67), *Pf*Exo RNase activity might result from exo- or endo-ribonuclease cleavage or a combination of both.

The wide substrate range of *Pf*Exo (dsDNA, 5′-flap, ssDNA, RNA–DNA) and its bipolarity on ssDNA and RNA–DNA hybrids suggests that the protein represents a unique exonuclease. Comparison with characterized exonucleases across genera does not assign it to a particular class as other reported bipolar exonucleases are specific for ssDNA or dsDNA (Supplementary Table S1). In terms of substrate specificity, *Pf*Exo seems closest to eukaryotic Exo I, but the latter has only 5′-3′ directionality on ssDNA and RNA–DNA hybrids.

**Figure 3.**
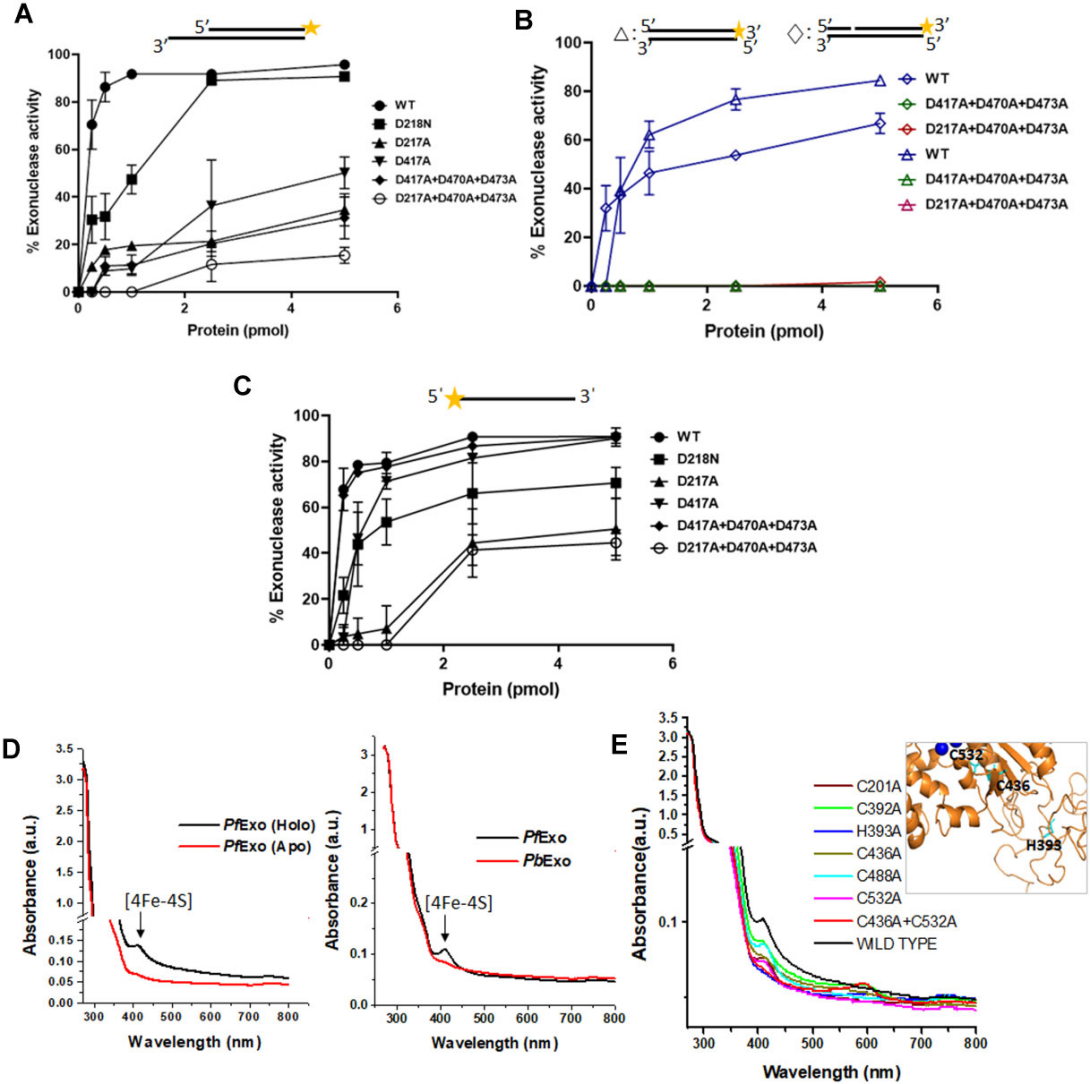
Active site and [Fe–S] coordination mutants of *Pf*Exo. Comparison of 5′-3′ exonuclease activity of *Pf*Exo active-site mutants on 5′-recessed dsDNA (**A**), blunt-end and 1 nt-gapped DNA (**B**), and 3′-5′ exonuclease activity on ssDNA **(C)**. Percent exonuclease activity was calculated as mean and standard deviation from three separate titration experiments. (**D**) UV–visible spectral scan of holo- and apo-forms of *Pf*Exo and *Pb*Exo with the [4Fe–4S] peak at 420 nm. (**E**) UV–visible scans of wild type *Pf*Exo and its cysteine and histidine mutants. Inset, *Pf*Exo structure model indicating positions of H393, C436 and C532. H393 lies within the insertion sequence (loop) and its actual position in the protein structure is unclear.

We generated site-directed mutants of *Pf*Exo based on conservation of active site aspartate residues spanning the three metal binding sites in *Ms*FenA (50). The 5′-flap endonuclease activity was completely abolished in *Pf*ExoD217A, *Pf*ExoD218N and *Pf*ExoD417A (Supplementary Figure S6A) suggesting that that disruption of either M1/M2/or M3 causes abolition of 5′-flap cleavage. While there was a decrease in 5′-3′ exonuclease activity on recessed substrate in the single and triple mutants (Figure 3A, Supplementary Table S5), complete abolition of activity was observed on blunt-end and 1 nt-gapped dsDNA (Figure 3B). The results indicated that *Pf*Exo loses activity when three active site residues (D217-D470-D473) contributing to all the three metal binding sites are mutated (Supplementary Table S5). Interestingly, 3′-5′ exonuclease activity on ssDNA was not abolished in any mutant and was maximally inhibited to only ∼40–46% in *Pf*ExoD217A and *Pf*ExoD217A-D470A-D473A (Figure 3C). Additionally, *Pf*ExoD217A-D470A-D473A completely lacked RNase activity on RNA–DNA hybrids in either polarity further confirming it as an intrinsic activity of *Pf*Exo (Supplementary Figure S6B).

### [4Fe–4S] cluster on *Pf*Exo is coordinated by a critical residue in the insertion sequence

A ssDNA-specific bipolar exonuclease, human EXO5, carries an iron-sulfur cluster which is important for its catalytic activity (68). Since the apicoplast has an active SUF pathway for [Fe–S] biogenesis (55), we checked the [Fe–S] status of *Pf*Exo by UV-visible scanning. A prominent peak at 420 nm indicated that the protein has a [4Fe–4S] cluster which could be removed upon chemical conversion to the apo-form (Figure 3D). Since the *P. berghei* ortholog lacks the insertion sequence but has 56.4% identity in the rest of the protein sequence, we expressed and purified recombinant *Pb*Exo (Supplementary Figure S7A) and also checked its [Fe–S] status. Surprisingly, *Pb*Exo lacked a [4Fe–4S] signal (Figure 3D) and thus differed from *Pf*Exo in terms of a post-translational modification.

In order to identify critical residues for coordination of the [4Fe–4S] on *Pf*Exo, we mutated all five cysteines (C201A, C392A, C436A, C488A, C532A) and a histidine (H393A). Individual mutations of C436A and C532A resulted in reduction of the [4Fe–4S] peak with complete loss in the double mutant (C436A + C532A) (Figure 3E). Additionally, the H393A mutant alone also resulted in complete loss of the [4Fe–4S] peak, indicating that these three residues are required for coordination of the iron-sulfur cluster. The fourth residue remains to be identified. Interatomic distance measurement in the *Pf*Exo molecular structure model gives the distance between C436 and C532 as between 16.2 and 19.3 Å which can form a pocket for coordination of a [4Fe–4S] cluster (Figure 3E). H393 lies in the disordered insertion sequence and its structural location cannot be reliably determined in the model.

### *Pb*Exo has a narrower substrate range than *Pf*Exo and is unipolar on ssDNA

**Figure 4.**
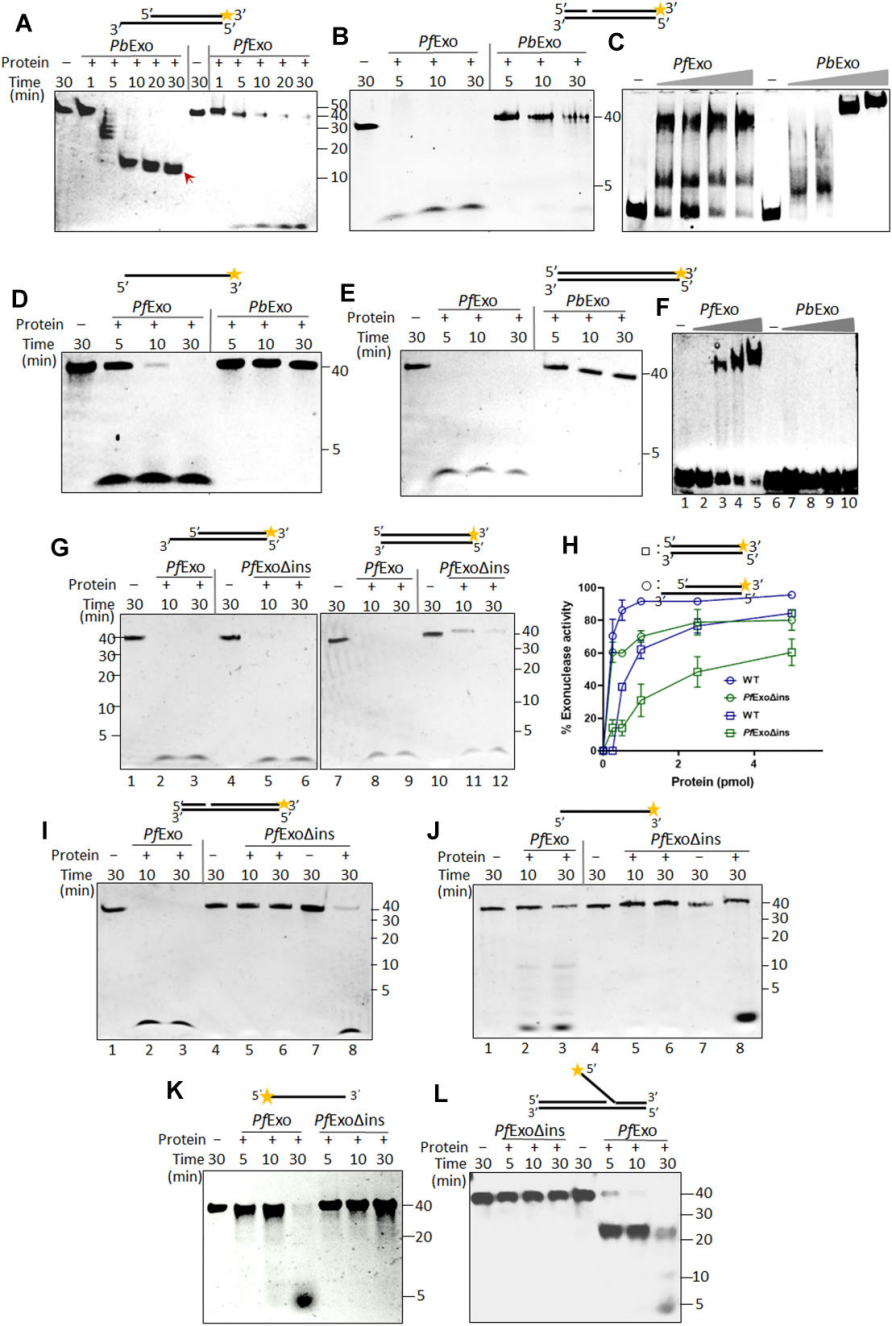
*Pb*Exo and *Pf*ExoΔins have a restricted substrate repertoire compared to *Pf*Exo. (**A**) terminal products of different lengths obtained after exonuclease cleavage of 5′-recessed DNA by *Pb*Exo and *Pf*Exo. The *Pb*Exo reaction product of ∼12 nt is indicated by a red arrow. Cleavage of 1 nt-gapped DNA (**B**), and EMSA with increasing protein concentrations (1, 2.5, 5 and 10 μM) to detect interaction of *Pf*Exo and *Pb*Exo with the substrate (**C**). *Pb*Exo does not cleave ssDNA in the 5′-3′ direction (**D**) and is not active on blunt-end DNA (**E**). (**F**) EMSA to detect interaction with blunt-end DNA reveals absence of DNA-protein complex formation by *Pb*Exo. 1, 2.5, 5 and 10 μM of *Pf*Exo and *Pb*Exo were used in binding reactions. (**G**) Comparison of 5′-3′ exonuclease activity of *Pf*Exo and *Pf*ExoΔins on 5′-recessed and blunt-end dsDNA substrates. (**H**) Graph comparing exonuclease activity of *Pf*Exo and *Pf*ExoΔins on 5′-recessed and blunt-end dsDNA. Circles and squares represent activity on 5′-recessed and blunt-end substrates, respectively. Comparison of 5′-3′ exonuclease cleavage on 1 nt-gapped dsDNA (**I**) and ssDNA (**J**), and 3′-5′ exonuclease on ssDNA (**K**) by *Pf*Exo and *Pf*ExoΔins. Cleavage of 5′-recessed DNA by *Pf*ExoΔins (lanes 7–8 in I and J) served as control for enzyme activity. (**L**) Comparison of 5′-flap cleavage by *Pf*ExoΔins and *Pf*Exo. All EMSAs and nuclease assays were repeated at least three times with different enzyme preparations.

To address whether sequence differences and the lack of [4Fe–4S] in *Pb*Exo alters its function compared to *Pf*Exo, activity of the former was tested on the diverse substrates. Although *Pb*Exo efficiently cleaved 5′-recessed dsDNA (with no activity on 3′-recessed dsDNA), the size of the terminal reaction product was ∼12 nt compared to 1 nt product generated by *Pf*Exo (Figure 4A, Supplementary Figure S7B, Table 1). It cleaved 1 nt-gapped DNA very poorly and was inactive on blunt-end DNA (Figure 4B and E). Unlike *Pf*Exo, the *P. berghei* ortholog cleaved ssDNA only in the 3′-5′ polarity but with lower efficiency and only at pH 9–9.5 (Figure 4D, Supplementary Figure S7C). *Pb*Exo was thus unipolar on recessed dsDNA and ssDNA, exhibiting opposite polarity on these substrates (Table 1). However, its 3′-5′ ssDNA activity at high pH may not be physiologically relevant. The 5′-flap cleavage was conserved in *Pb*Exo with ∼25 nt product being generated (Supplementary Figure S7D). On RNA–DNA hybrids, *Pb*Exo cleaved RNA in both directions, but with low efficiency in the 3′-5′ polarity (Supplementary Figure S7E and F, Table 1). As observed with its activity on 5′-recessed dsDNA, the terminal RNA cleavage product was also ∼12 nt (Supplementary Figure S7E).

**Table 1.** Comparison of activities of *Pf*Exo, *Pb*Exo, *Pf*ExoΔins and *Pb*Exo-ins^+^ on DNA and RNA–DNA substrates

Substrate/activity	*Pf*Exo	*Pb*Exo	*Pf*ExoΔins	*Pb*Exo-ins^+^
**dsDNA, 5′-3′exonuclease:**				
• **5′-recessed**	+++ (1 nt product)	+++ (∼12 nt product)	++ (1 nt product)	+++ (1 nt product)
• **Blunt-end**	+++	–	++	+++
• **1nt-gapped**	+++	+	–	++
**dsDNA, 3′-5′ exonuclease**	–	–	ND	ND
**ssDNA, 5′-3′exonuclease**	+++ (1 nt product)	–	–	+++
**ssDNA, 3′-5′exonuclease**	++ (∼5 nt product); pH 6–9.5	+ (∼5 nt product); pH 9–9.5	–	++ (5 nt product); pH 6–9.5
**5′Flap, endonuclease**	+++ (∼25 nt flap + lower products)	+++ (∼25 nt flap product)	–	+++ (∼25 nt flap + lower products)
**RNA–DNA hybrid**, **5′-3′ RNase**	+++ (5 nt major product)	+++ (∼12 nt product)	+++ (5 nt product)	ND
**RNA–DNA hybrid**, **3′-5′ RNase**	+++ (1 nt product)	+ (1 nt product)	+++ (1 nt product)	ND

ND, not determined.

To understand whether the compromised *Pb*Exo activity on 1 nt-gapped dsDNA and its inability to cleave blunt-end DNA was a result of inert DNA-protein complex formation with these substrates or compromised DNA binding, we carried out EMSAs to detect complex formation. A clear shift was seen with *Pb*Exo and 1 nt-gapped dsDNA as probe, although at concentrations higher than *Pf*Exo (Figure 4C), partly explaining its poor hydrolysis of the substrate. On the other hand, in contrast to *Pf*Exo, *Pb*Exo was unable to bind blunt-end dsDNA even at high concentrations (Figure 4F) indicating that its inability to cleave the substrate results from lack of interaction with blunt-end DNA.

### The large insertion in *Pf*Exo is a determinant of its expanded substrate range

To explore protein features which functionally distinguish the *P. falciparum* and *P. berghei* Exo orthologs, we first tested the effect of the [4Fe–4S] cluster on *Pf*Exo activity by comparing the holo- and apo-forms of the protein in cleavage assays. The apo-form behaved identically to the holo-protein when tested for exonuclease activity on 5′-3′ recessed dsDNA and 3′-5′ and 5′-3′ ssDNA as well endonucleolytic cleavage of the 5′-flap (Supplementary Figure S8) indicating that the iron-sulfur cluster does not contribute to *in vitro* catalysis by *Pf*Exo.

All critical aspartate and glutamate residues at the active site are conserved between *Pf*Exo and *Pb*Exo. One distinguishing feature of *Pf*Exo and some of its Laveranian orthologs, such as *P. reichenowi* and *P. blacklocki*, is the presence of an aspartate (D218) adjacent to the active site residue D217; *Pf*Exo D218 is replaced by an asparagine in other *Plasmodium* species including *P. berghei* (Supplementary Figure S3). The *Pf*Exo D218N mutation caused only minor reduction in exonuclease activity on the panel of dsDNA and ssDNA substrates tested (Figure 3A, C; Supplementary Figure S9) indicating that it is not a critical residue distinguishing *Pf*Exo and *Pb*Exo.

Sequence alignment of *Pf*Exo with its orthologs in other *Plasmodium* species showed that a major part of the *Pf*Exo large central insertion was conserved in species of the subgenus *Laverania* but was absent in the rest, including *P. berghei* (Supplementary Figure S3A). We thus created a deletion of this 153 aa stretch to generate *Pf*ExoΔins (Supplementary Table S2). Removal of the insertion destabilized the protein considerably, hence all activity and binding assays were performed immediately upon protein purification. When purified *Pf*ExoΔins (Supplementary Figure S10A) was assayed, it was seen to retain the ability to cleave 5′-recessed dsDNA, had reduced activity on the blunt-end substrate (Figure 4G, H) but was not active on 1 nt-gapped dsDNA (Figure 4I) (Table 1). Moreover, it was unable to cleave ssDNA in the 5′-3′ polarity (Figure 4J) and had negligible activity on ssDNA in the 3′-5′ polarity (Figure 4K). Thus, the loss of the insertion altered the substrate repertoire of *Pf*Exo; its ability to hydrolyze ssDNA in either direction was compromised as in the *P. berghei* ortholog. Surprisingly, however, *Pf*ExoΔins completely lost 5′-flap endonuclease activity (Figure 4L) suggesting that the insertion makes a crucial contribution to DNA substrate recognition/cleavage. However, unlike *Pb*Exo which exhibited lower efficiency on recessed 3′-5′ RNA–DNA hybrid, *Pf*ExoΔins behaved like wild-type *Pf*Exo on RNA–DNA hybrid substrates. The terminal RNA product lengths obtained with *Pf*Exo and *Pf*ExoΔins were also identical (Supplementary Figure S10B; Figure 2H, I; Table 1)

### The *Pf*Exo insertion element is crucial for successful interaction with diverse DNA substrates

**Figure 5.**
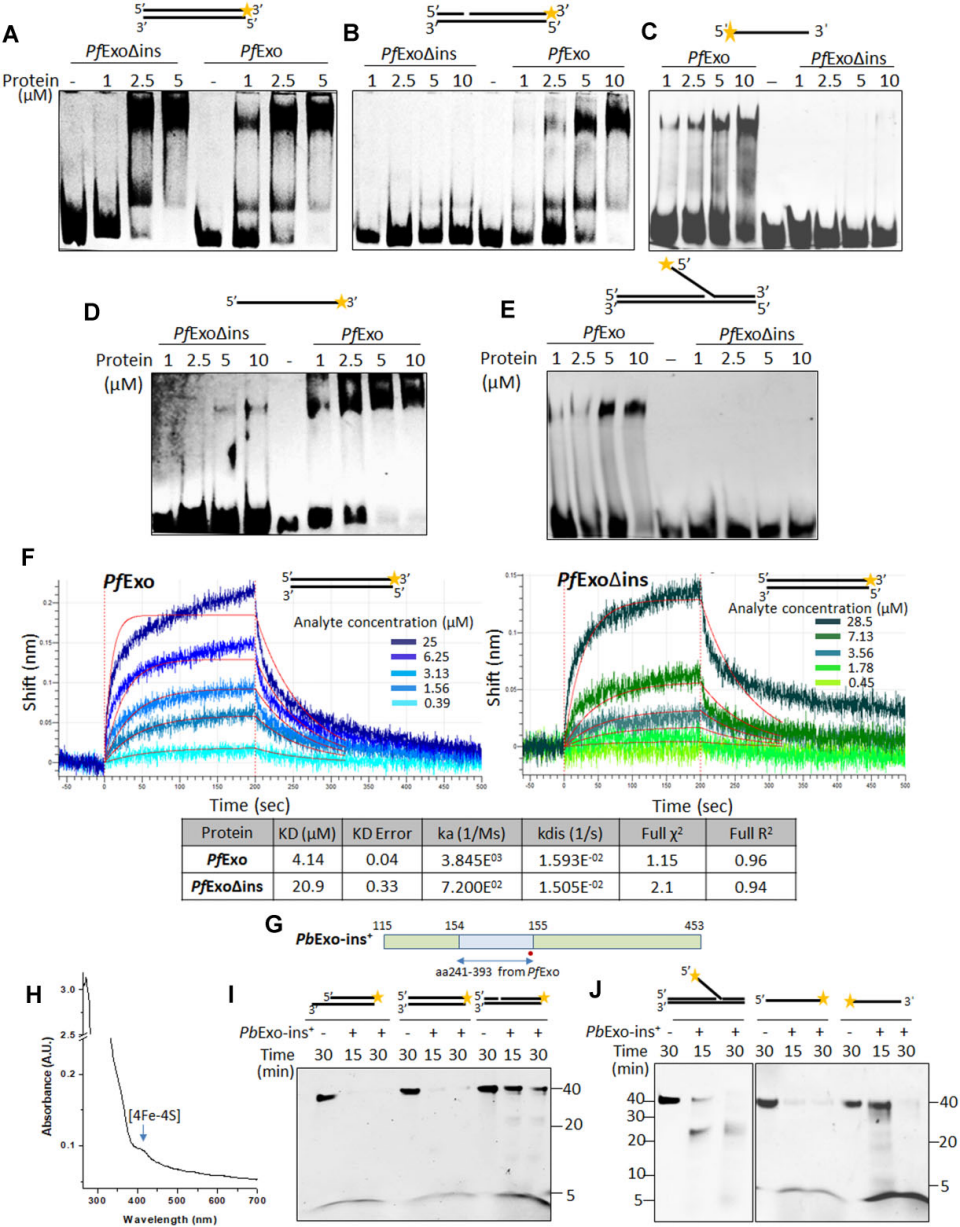
The *Pf*Exo insertion sequence increases affinity of the protein for DNA and its incorporation into *Pb*Exo diversifies the substrate range of the latter. EMSAs for comparison of DNA-binding of *Pf*Exo and *Pf*ExoΔins with blunt-end (**A**) and 1 nt-gapped (**B**) dsDNA substrates as probes. EMSA for comparison of protein interaction with 3′-5′ and 5′-3′ exonuclease ssDNA substrates (**C** and **D**, respectively). (**E**) EMSA to detect interaction of *Pf*Exo and *Pf*ExoΔins with 5′-flap DNA as probe. (**F**) BLI analysis to determine affinity of *Pf*Exo and *Pf*ExoΔins towards the blunt-end dsDNA substrate. Concentrations of analyte (*Pf*Exo or *Pf*ExoΔins) are indicated in the colour key. (**G**) Diagrammatic representation showing the position in *Pb*Exo where 153 amino acids comprising the *Pf*Exo insertion were introduced to generate *Pb*Exo-ins^+^. (**H**) UV-VIS scan showing that *Pb*Exo-ins^+^ gains [4Fe–4S] cluster. (**I**) Activity of *Pb*Exo-ins^+^ on 5′-recessed, blunt-end and 1 nt-gapped dsDNA. (**J**) 5′-flap endonuclease and exonuclease activity of *Pb*Exo-ins^+^ on ssDNA substrates in either polarity. All experiments were repeated at least two times with different enzyme preparations.

To address whether the reduced DNA substrate repertoire of *Pf*ExoΔins resulted from alteration of its DNA-binding, we carried out EMSAs to compare interaction of *Pf*Exo and *Pf*ExoΔins with DNA substrates on which the latter showed reduced or no exo/endonucleolytic cleavage. Both *Pf*ExoΔins and *Pf*Exo bound blunt-end DNA but DNA-protein shift with the former was seen at higher protein concentration (Figure 5A). The lower affinity of *Pf*ExoΔins for blunt-end DNA was confirmed by biolayer interferometry (BLI) with the protein showing ∼5-fold higher *K*_D_ than *Pf*Exo (Figure 5F). Moreover, *Pf*ExoΔins was unable to bind 1 nt-gapped dsDNA, a substrate that it cannot cleave (Figure 5B). *Pf*ExoΔins did not process the 1 nt-gapped substrate even though it offered an unlabelled blunt-end that the protein could possibly cleave to generate a 5′-recessed substrate which in turn could be its substrate (Figure 4G, I). EMSA with a 14 bp blunt-end DNA, as present in the gapped substrate, showed that it did not recognize blunt-end DNA of this size (Supplementary Figure S10C). Moreover, it also did not cleave a 1 nt-gapped substrate with a larger unlabelled dsDNA stretch of 44 nt present 5′ of the gap (Supplementary Figure S10D), suggesting that the primary determinant for recognition of a gapped substrate was its pre-bent DNA conformation (69); *Pf*Exo bound and cleaved substrates with this structure while *Pf*ExoΔins did not.

Faint DNA-*Pf*ExoΔins complexes were seen with both 3′-5′ and 5′-3′ ssDNA probes as compared to *Pf*Exo (Figure 5C and D) corresponding to the negligible or no cleavage of these substrates by *Pf*ExoΔins. The complete inability of *Pf*ExoΔins to cleave 5′-flaps was also reflected in the lack of binding to this substrate, whereas clear shifts were observed with *Pf*Exo (Figure 5E). Hence, the loss of the insertion gravely compromised the ability of the protein to interact with specific DNA substrates thus drastically reducing its substrate repertoire. Our results indicate a functional role for the insertion in *Pf*Exo. It remains to be seen whether the insertion region makes direct contacts with DNA or alters protein conformation such that specific substrates are accessed better by the active site.

### *Pb*Exo demonstrates *Pf*Exo-like properties upon acquiring the *Pf*Exo insertion element

To confirm the functional role of the LCR, we next introduced the *Pf*Exo insertion (153 residues) at the corresponding site in *Pb*Exo to generate *Pb*Exo-ins^+^ (Figure 5G; Supplementary Figure S11A). UV-VIS scan of purified *Pb*Exo-ins^+^ detected [4Fe–4S] (Figure 5H) further confirming the critical role of His393 within the insertion for Fe–S cluster coordination. *Pb*Exo-ins^+^ cleaved 5′-recessed dsDNA generating final cleavage products of 1 nt as observed for *Pf*Exo and unlike the ∼12 nt product generated by *Pb*Exo (Figure 5I; Figure 4A). In contrast to *Pb*Exo, *Pb*Exo-ins^+^ could also cleave blunt-end and 1 nt-gapped dsDNA (Figure 5I; Figure 4B, E). It processed 5′-flap and additionally generated lower cleavage products (Figure 5J) as it was now able to cleave ssDNA better in the 3′-5′ polarity at pH8.5 as compared to *Pb*Exo (Figure 5J; Supplementary Figure S7C and S11B). Further, *Pb*Exo-ins^+^ gained bipolarity on ssDNA and cleaved ssDNA in the 5′-3′ direction (Figure 5J). Incorporation of the *Pf*Exo insertion in *Pb*Exo thus conferred properties it had earlier lacked, thus confirming the role of the insertion element in DNA substrate diversification of the *P. falciparum* ortholog.

### *Pb*Exo is essential for survival of asexual parasites

**Figure 6.**
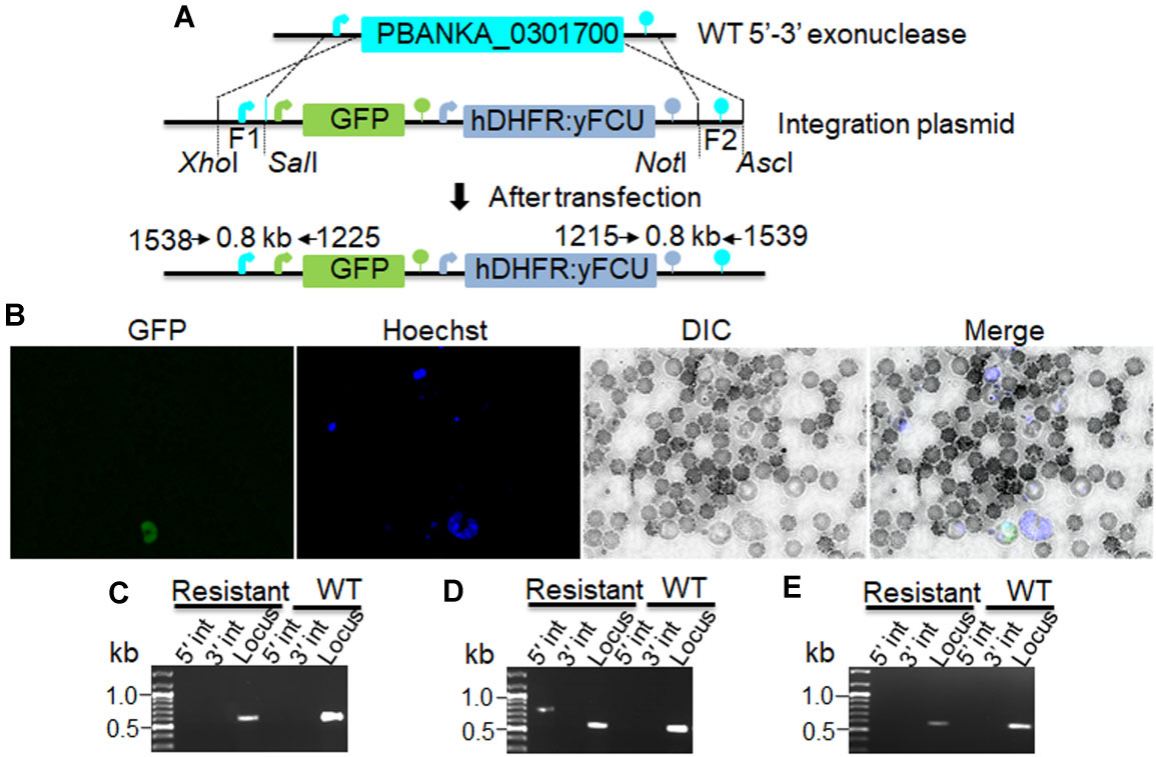
5′-3′ exonuclease is indispensable in *P. berghei* blood stages and interacts with 3′-5′ Exo/Pol domain of apicoplast *Pf*Prex. (**A**) Schematic showing the double crossover (DCO) homologous recombination strategy for the generation of 5′-3′ exonuclease knockout parasites. Genomic locus of the target gene is flanked by 5′ and 3′ UTRs (cyan arrow and lollipop, respectively). The integration plasmid consists of GFP and hDHFR cassette flanked by 5′ and 3′ UTRs of 5′-3′ exonuclease. GFP cassette consists of HSP70 promoter (green arrow), GFP ORF (green box), HSP70 3′ UTR (green lollipop) and hDHFR cassette consists of EF1α promoter (blue arrow), hDHFR ORF (blue box) and DHFR-TS 3′ UTR (blue lollipop). (**B**) GFP was observed in the recovered parasites. Parasite nucleus was stained with Hoechst 33342. **(C–E)** Diagnostic PCR revealed that double cross over recombination was not successful. No 5′ and 3′ integration bands were detected from recovered parasites genomic DNA. A band in 5′ integration PCR was observed in the third transfection. Gene locus (0.6 kb) was amplified using primer pair 1704/1705 in both WT and recovered parasites.

*PiggyBac* mutagenesis of *Pf*Exo with low mutagenesis index score and mutagenesis fitness score has indicated essentiality of the gene in *P. falciparum* (58). Since the *P. berghei* knockout has a reported slow growth phenotype (60), we planned disruption of *Pb*Exo to follow its effect in different stages of the parasite life cycle from the rodent host to mosquito. *Pb*Exo N-terminal region is also predicted to carry an apicoplast targeting sequence and is likely to be targeted to the organelle. We attempted to disrupt the gene in *P. berghei* by double cross-over homologous recombination (Figure 6A). The targeting cassette comprised 570 and 575 bp of the 5′ and 3′ UTRs respectively flanking a GFP reporter and hDHFR:yFCU selection marker. Four attempts to disrupt the function of *Pb*Exo by generating a direct knockout were not successful. In the first attempt, the parasites were not recovered after drug selection. In the second, third and fourth attempts parasites were recovered after drug selection and transfection efficiency was 1.1 × 10^−10^, 1.0 × 10^−9^ and 9.8 × 10^−10^ respectively. Whereas, the transfection efficiency of a control plasmid that successfully targeted a non-essential locus was 4.7 × 10^−5^. Recovered parasites were negative for GFP except after third transfection a rare GFP was observed (Figure 6B). To examine whether the targeting cassette was integrated at the 5′-3′ exonuclease locus, we amplified the region by using primers which were designed to bind beyond recombination region. No fragments were amplified by this PCR except after third transfection a band in 5′ integration PCR was observed (Figure 6C–E). The exonuclease locus was amplified in all PCRs suggesting that enough wild-type population was present during harvesting of the parasites. The lower transfection efficiency observed in *PbExo* was probably due to non-survival of the knockout parasites. We hypothesize that parasites lacking *Pb*Exo failed to grow in the subsequent cycle. This gene has also shown a significantly slow growth phenotype in a barcoded *P. berghei* knockout mutant (60) although a detailed study would be required to confirm the phenotype. Our results indicate that the 5′-3′ exonuclease is possibly essential for blood stage propagation.

### *Pf*Exo interacts with the apicoplast atypical DNA polymerase *Pf*Prex

**Figure 7.**
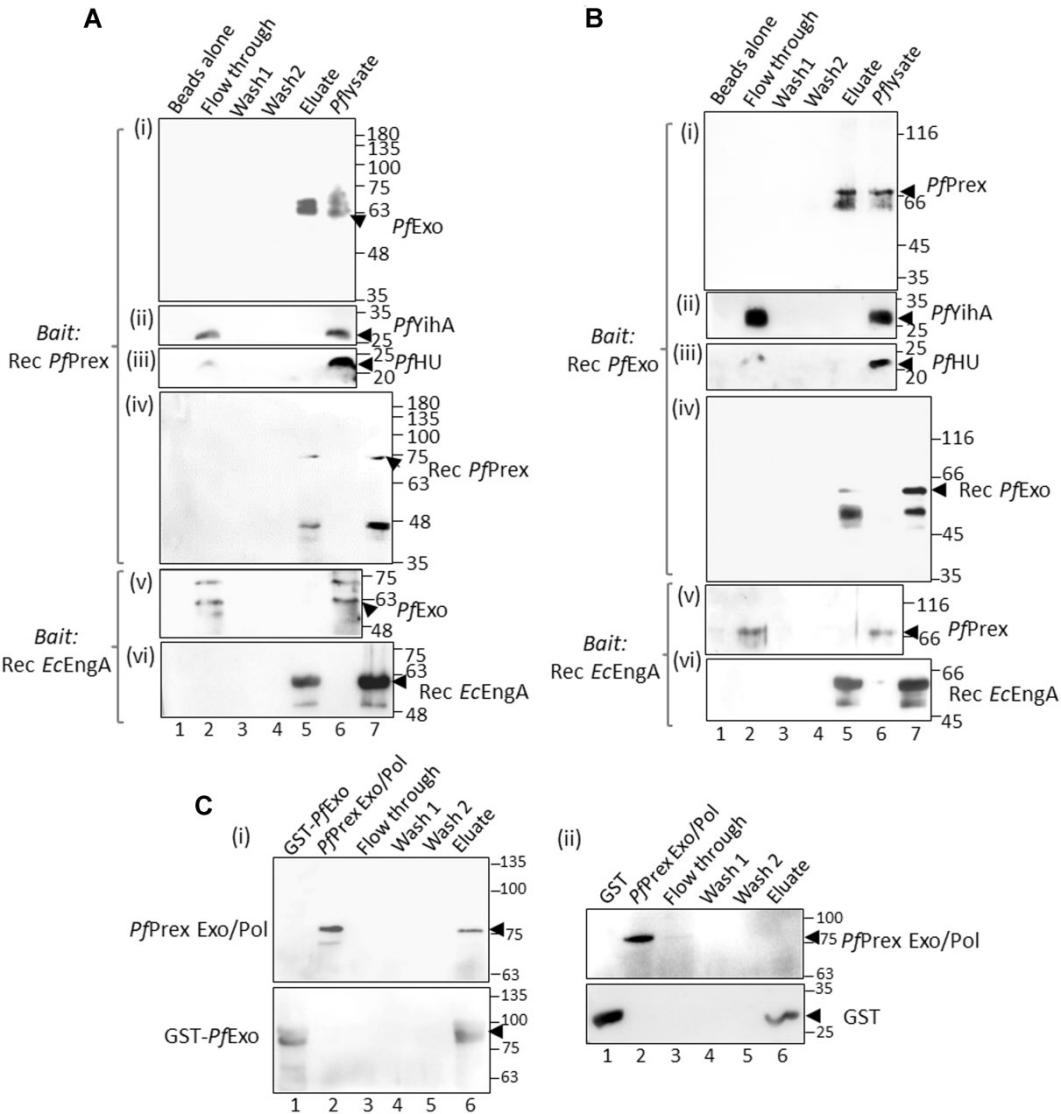
(**A**) Experiment using recombinant *Pf*Prex Exo/Pol domain (Rec *Pf*Prex) bound to Ni-NTA beads as bait to pull-down interacting protein(s) from the parasite lysate. *Pf*Exo was specifically detected in the eluate after western blotting with anti-*Pf*Exo sera (panel i, lane 5); no *Pf*Exo signal was seen when recombinant *Ec*EngA (Rec *Ec*EngA) was used as the negative control bait protein (panel v, lane 5). The unrelated proteins, apicoplast-targeted *Pf*YihA (detected using anti-*Pf*YihA sera) and *Pf*HU (detected using anti-*Pf*HUp sera), were not pulled down by Rec *Pf*Prex (panels ii and iii, lane 5). Western blots probed with anti-6X His Ab detected the bait proteins Rec *Pf*Prex (panel iv) and Rec *Ec*EngA (panel vi) in the respective eluate fractions. Lane 7 in panels iv and vi is Rec *Pf*Prex and Rec *Ec*EngA, respectively, loaded as marker. (**B**) Recombinant *Pf*Exo (Rec *Pf*Exo) bound to Ni-NTA beads used to pull-down protein(s) from the parasite lysate. Western blots using anti-*Pf*Prex Exo/Pol, anti-*Pf*YihA and anti-*Pf*HUp sera specifically detected *Pf*Prex in the eluate fraction (panel i, lane 5), with no signals observed for *Pf*YihA and *Pf*HUp in the eluate (panels ii and iii). Western blots probed with anti-6XHis Ab detected the bait proteins Rec *Pf*Exo (panel iv) and Rec *Ec*EngA (panel vi) in the eluate fraction. Lane 7 in panels iv and vi is Rec *Pf*Exo and Rec *Ec*EngA, respectively. The pull-down assays were repeated three times. (**C**) In vitro interaction of GST-*Pf*Exo and *Pf*Prex Exo/Pol using GST-*Pf*Exo bound to glutathione agarose beads as bait (i). GST alone served as control bait (ii). Western blot with anti-6X His and anti-GST Abs detected co-elution of *Pf*Prex Exo/Pol and GST-*Pf*Exo, respectively in the eluate fraction (lane 6) in (i). *Pf*Prex Exo/Pol did not interact with GST alone (ii). Lanes 1 and 2 have purified proteins loaded as markers. The experiment was repeated two times.

Since DNA replication requires Flap endonuclease and RNaseH functions which are harboured by *Pf*Exo, the protein is a candidate constituent of the plDNA replication machinery. The possibility of its interaction with the apicoplast DNA polymerase *Pf*Prex or its proteolytically matured Exo/Pol domain was thus tested (17). Recombinant *Pf*Prex Exo/Pol domain (Supplementary Figure S12A, B) bound to Ni-NTA beads was used as bait in pull-down experiments. *Pf*Exo was specifically detected in the imidazole eluate (Figure 7A, panel i, lane 5). It did not bind to beads alone (Figure 7A, panel i, lane 1), or to *Ec*EngA used as control bait protein (Figure 7A, panel v, lane 5). The unrelated apicoplast-targeted protein *Pf*YihA and apicoplast DNA organization protein *Pf*HU were not detected in the eluate (Figure 7A, panels ii and iii, lane 5) indicating specific interaction of *Pf*Exo with *Pf*Prex Exo/Pol. Similarly, when recombinant *Pf*Exo was used as bait, it specifically pulled down *Pf*Prex Exo/Pol (detected by anti-*Pf*Prex Exo/Pol serum; Supplementary Figure S12C) from the parasite lysate (Figure 7B, panel i, lane 5). Other apicoplast proteins (*Pf*YihA and *Pf*HU) were not pulled down by *Pf*Exo (Figure 7B, panels ii and iii), and there was no *Pf*Prex Exo/Pol signal in the eluate from the control bait (*Ec*EngA) set (Figure 7B, panel v). To further confirm the interaction between *Pf*Exo and *Pf*Prex Exo/Pol, purified recombinant GST-tagged *Pf*Exo, or GST alone, bound to glutathione agarose beads were used to pull down purified *Pf*Prex Exo/Pol. Interaction of *Pf*Prex Exo/Pol was detected only with GST-*Pf*Exo with no signal observed in eluates when GST alone was used as bait (Figure 7C). The *Pf*Exo-*Pf*Prex interaction is suggestive of an important role for *Pf*Exo in apicoplast replication and organelle genome maintenance.

## Discussion

Our results identify an exonuclease of the human malaria parasite *P. falciparum* as an apicoplast-targeted enzyme capable of processing multiple substrates for DNA and RNA–DNA transactions. The DNase and RNase activities of *Pf*Exo and those of its ortholog from the rodent malaria species *P. berghei* suggest their important role in apicoplast genome replication and repair. The inability of *Pb*Exo knockout parasites to survive and grow indicates an essential role for the protein. Additionally, significant differences in substrate recognition between *Pf*Exo and *Pb*Exo suggest the possibility of altered functional roles in the two *Plasmodium* species. Although both orthologs functioned comparably as 5′-flap endonuclease and as 5′-3′ exonuclease on recessed dsDNA (Table 1), *Pf*Exo had superior activity on 1 nt-gapped dsDNA and additionally processed blunt-end dsDNA. *Pf*Exo also exhibited bipolarity (as 5′-3′ and 3′-5′ exonuclease) on ssDNA and as a ribonuclease on RNA–DNA hybrids. On the other hand, the 3′-5′ exonuclease and ribonuclease activity residing in *Pb*Exo was lower on both ssDNA and RNA–DNA hybrids, respectively, and it completely lacked 5′-3′ exonuclease activity on ssDNA.

The enriched *P. falciparum* apicoplast proteome (70) does not identify a FEN, and the only identified apicoplast exoribonuclease (PF3D7_1106300) is a homolog of yeast 5′-3′ ribonuclease 2 implicated in RNA quality control (71)*. Pf*Exo is thus likely to be the only apicoplast-targeted protein with a metal-ion coordinated protein fold similar to FENs and T4 RNaseH. Its 5′-flap removal activity and association with the apicoplast *Pf*Prex suggests a role in plDNA replication for removal of Okazaki fragments on the lagging strand. Since *Pf*Prex is an atypical PolA that has lost the 5′-3′ exonuclease domain (31), the 5′-3′ RNaseH activity of *Pf*Exo could take over removal of ribonucleotide primers from Okazaki fragments. Interaction of *Pf*Exo with *Pf*Prex also suggests the possibility of coupling 5′-3′ DNA exonuclease to nick translation synthesis on nicked/gapped substrates (72). *Pf*Exo has exonucleolytic hydrolysis activity on blunt-end DNA which is conserved in FENs (73) but this is lost in *Pb*Exo (Table 1). During plDNA replication, *Pf*Prex can incorporate ribonucleotides which need to be removed by a 3′-5′ RNase (20). Although *Pf*Prex exhibits 3′-5′ RNase activity, measurement of polymerization/excision rates have indicated that not all misincorporated rNTPs are excised leading to introduction of ∼10 rNTPs per replication cycle (20). The *Pf*Exo 3′-5′ RNase activity could supplement rNTP proof-reading by *Pf*Prex to minimize misincorporation; since 3′-5′ RNase activity on RNA–DNA hybrids is poor in *Pb*Exo, this function might fully reside in the *P. berghei* apicoplast polymerase.

*Pf*Exo/FEN is unique in possessing unipolar (5′-3′) exonuclease activity on dsDNA combined with bipolarity on both ssDNA and RNA–DNA substrates. As in human ExoV, bipolarity of *Pf*Exo on ssDNA might allow it to process ssDNA on stalled replication forks, thus contributing to replication fork restart (74) and/or degrade ssDNAs containing deaminated and methylated bases as in *Thermus thermophilus* Exo I (75). Three-stranded DNA-RNA hybrids (R-loops) generated co-transcriptionally in cells can be a genotoxic obstacle and inhibit replication fork movement. The resolution of R-loops, as with human RNaseH2 (76), is another possible function of *Pf*Exo. The role of *Pf*Exo in DNA repair via long-patch BER and/or mismatch repair (MMR) via its FEN and 5′-3′ exonuclease activities, respectively cannot be ruled out. Between the *P. falciparum* apicoplast and mitochondrion, most BER enzymes seem to target to the latter (30,32). An MMR MutS homolog is detected in the apicoplast proteome (70), although apicoplast MutL or MutH homologs are not identified. If MMR operates in the apicoplast, *Pf*Exo could function in exonucleolytic DNA cleavage upstream of the mismatched base in Exo I-dependent MMR or as a FEN in Exo I-independent MMR subpathway (77).

*Pf*Exo reveals a novel functional implication of protein insertions in *P. falciparum* where the sequence confers unique DNA substrate specificities as compared to the *P. berghei* ortholog lacking the insertion. Although unique protein insertion sequences which are conserved across *Plasmodium* species have been implicated in protein activity and stability (78–80), roles of LCRs unique to *P. falciparum* have thus far been proposed in host immunity (5–7), as tRNA sponges (8) or as recombination hotspots (9). The *Pf*Exo insertion mediates DNA-recognition on 5′-flap and 1 nt-gapped dsDNA and enhances binding to blunt-end and ssDNA, as inferred from analyses of *Pf*ExoΔins (Table 1). The retention of comparable 5′-flap endonuclease activity in *Pb*Exo suggests that residues outside the insertion compensate for its absence in the *P. berghei* ortholog. The *Pf*Exo insertion is also a determinant of bipolar ssDNA cleavage, activities that are lost in *Pf*ExoΔins, and thus brings about a combination of properties that distinguish *Pf*Exo from other known exonucleases (Supplementary Table S1). Significantly, introduction of the *Pf*Exo insertion in *Pb*Exo (*Pb*Exo-ins^+^) caused it to gain 5′-3′ exonuclease function on both blunt-end and ssDNA and confirmed the role of the insertion sequence in mediating substrate-specific activity. The narrow pH range (9–9.5) for the *Pb*Exo 3′-5′ exonuclease activity was also broadened to physiologically relevant pH in *Pb*Exo-ins^+^ (Table 1). Our *Pf*Exo molecular structure model did not predict structural folds for the insertion. The AlphaFold Protein Structure Database (PDB: AF-096139-F1; https://alphafold.ebi.ac.uk/) has the *Pf*Exo insertion modelled as large alpha helices with interspersed loops with low to very low confidence (Supplementary Figure S13); both structure models predict identical folds for the conserved domains. The presence of extruded loops of the insertion sequence in the *Pf*Exo models precludes the possibility of generating a reliable *in silico* prediction model for *Pf*Exo-*Pf*Prex Exo/Pol interaction. Knowledge of the precise structural implications of the insertion in intra- and inter-domain interactions and how it alters DNA binding and subsequent catalysis will depend on resolution of *Pf*Exo structure in complex with diverse nucleic acid substrates.

Distinct from other *Plasmodium* species, LCRs in the A + T-rich *P. falciparum* genome have been broadly classified into three families based on their sequence (9) ‘heterogenous’, with a reduced set of amino acids, relative high A + T content and low diversity among *P. falciparum* isolates; ‘PolyN’, with a predominance of Asn repeats and high level of diversity; and ‘High GC’, with relatively low A + T content and high diversity. The large *Pf*Exo insertion is GC-rich (30% G + C in the insertion compared to 19% G + C in rest of the *Pf*Exo coding sequence), has relatively low disorder, contains negatively charged stretches, and diversity among *P. falciparum* isolates is primarily limited to the number of NDHT repeats (4–7 repeats) in the middle of the insertion. All Laveranian *Plasmodium* species carrying the insertion have the conserved His residue (His393 in *Pf*Exo) critical for [4Fe–4S] complexation (Supplementary Figure S3) and are thus likely to be the only orthologs containing [4Fe–4S]. The redox properties of [Fe–S] clusters on DNA processing and repair proteins have been proposed to electronically probe DNA integrity *in vivo* by DNA-mediated charge transport (DNA CT) (81), and DNA CT has been further proposed to change [Fe–S] oxidation states to alter the conformation, activities and interactions of their respective DNA-interacting enzymes (82,83). Comparable activity of apo- and holo-*Pf*Exo *in vitro* indicates that [4Fe–4S] is not directly involved in catalysis but could play a regulatory role in DNA-protein interactions within the apicoplast. Bound [4Fe–4S] might also aid the mildly intrinsically disordered *Pf*Exo insertion domain in adopting greater conformational stability upon DNA binding to specific substrates.

Single mutations of selected *Pf*Exo aspartate residues (D217A, D218N or D417A) corresponding to those implicated in carboxylate contacts with metal ions at the active site in *Ms*FenA (50) abolished 5′-flap cleavage. However, the 5′-3′ exonuclease activity on recessed dsDNA was reduced only mildly in *Pf*ExoD218N, reduced to 30–40% in *Pf*ExoD217A and *Pf*ExoD417A, with maximal reduction (∼12% activity) observed in the triple mutant (*Pf*ExoD217A-D470A-D473A) which would affect all three metal binding sites (Supplementary Table S5). The altered effects of single aspartate mutants on 5′-flap cleavage and 5′-3′ exonuclease on recessed DNA suggests nuanced differences in recruitment and cleavage of different substrates. Cleavage of 1 nt-gapped and blunt-end DNA was more sensitive to triple mutations (*Pf*ExoD217A-D470A-D473A and *Pf*ExoD417A-D470A-D473A) compared to recessed DNA. Interestingly, none of the five *Pf*Exo mutants abolished activity on ssDNA in 3′- 5′ polarity; maximal reduction (∼40–46% activity) in the single- and triple- mutant carrying D217A indicated that the M1 coordination complex, and not M2 or M3, has a significant role in 3′-5′ ssDNA cleavage. Contribution to ssDNA 3′-5′ exonuclease might also come from outside M1/M2/M3 active site in *Pf*Exo. The abolition of ssDNA exonuclease function in *Pf*ExoΔins despite substrate engagement (albeit lower compared to *Pf*Exo), appearance of 5′-3′ ssDNA exonuclease activity and expansion of the active pH range of 3′-5′ ssDNA exonuclease in *Pb*Exo-ins^+^ (Table 1) points to an important role for the insertion sequence in mediating ssDNA cleavage in either polarity. Support for the role of the insertion in ssDNA hydrolysis also comes from the fact that *Pb*Exo has all the conserved active site asparate residues (except D218), yet is a highly compromised exonuclease on ssDNA. The insertion element in *Pf*Exo is rich in aspartate and glutamate residues (Supplementary Figure S3A); the possibility of a combination of these engaging a metal ion outside the main active site which contributes to ssDNA hydrolysis cannot be ruled out.

Our findings provide evidence that *Pf*Exo is a novel FEN/Exo with additional bipolarity on ssDNA and RNA–DNA hybrids with a role in parasite growth and survival. The contribution of its unique LCR insertion to DNA processing identifies a new role for *P. falciparum* LCR elements in enzyme function. The LCR also contributes to interspecific functional differences between *Pf*Exo and its ortholog in *P. berghei* by altering DNA binding to specific substrates *in vitro* and possibly contributing to [4Fe–4S]-mediated interactions with DNA *in vivo*. *Pf*Exo's interaction with *Pf*Prex provides support to the surmise that its flap-cleavage and 5′-3′ exonuclease functions could complement *Pf*Prex 3′-5′ Exo/Pol during plDNA replication. Although awaiting experimental validation, the absence of another predicted 5′-flap endonuclease and RNaseH in the apicoplast also positions *Pf*Exo as a candidate protein for BER/MMR-mediated DNA repair.

## Supplementary Material

gkae512_Supplemental_Files

## Data Availability

All relevant data are available in the main manuscript and supplementary information. The *Pf*Exo structure homology model is available in ModelArchive at https://www.modelarchive.org/doi/10.5452/ma-dkahf.
