# Supplementary material for: A Plasmodium apicoplast-targeted unique exonuclease/FEN exhibits interspecies functional differences attributable to an insertion that alters DNA-binding
Source: Nucleic Acids Res. 2024 Jun 18;52(13):7843–62. doi: 10.1093/nar/gkae512 (PMC11260460; doi:10.1093/nar/gkae512)
Supplement: gkae512_Supplemental_Files [file gkae512_supplemental_files.zip › Supplementary Figure S1.pdf]

Supplementary Data

A *Plasmodium* apicoplast-targeted unique exonuclease/FEN exhibits interspecies functional differences attributable to an insertion that alters DNA-binding

Tribeni Chatterjee[1#], Anupama Tiwari[1#], Ritika Gupta[1], Himadri Shukla[2], Aastha Varshney[2], Satish Mishra[2], Saman Habib[1*]

[1]Division of Biochemistry and Structural Biology, CSIR-Central Drug Research Institute, Lucknow, India

[2]Division of Molecular Microbiology and Immunology, CSIR-Central Drug Research Institute, Lucknow, India

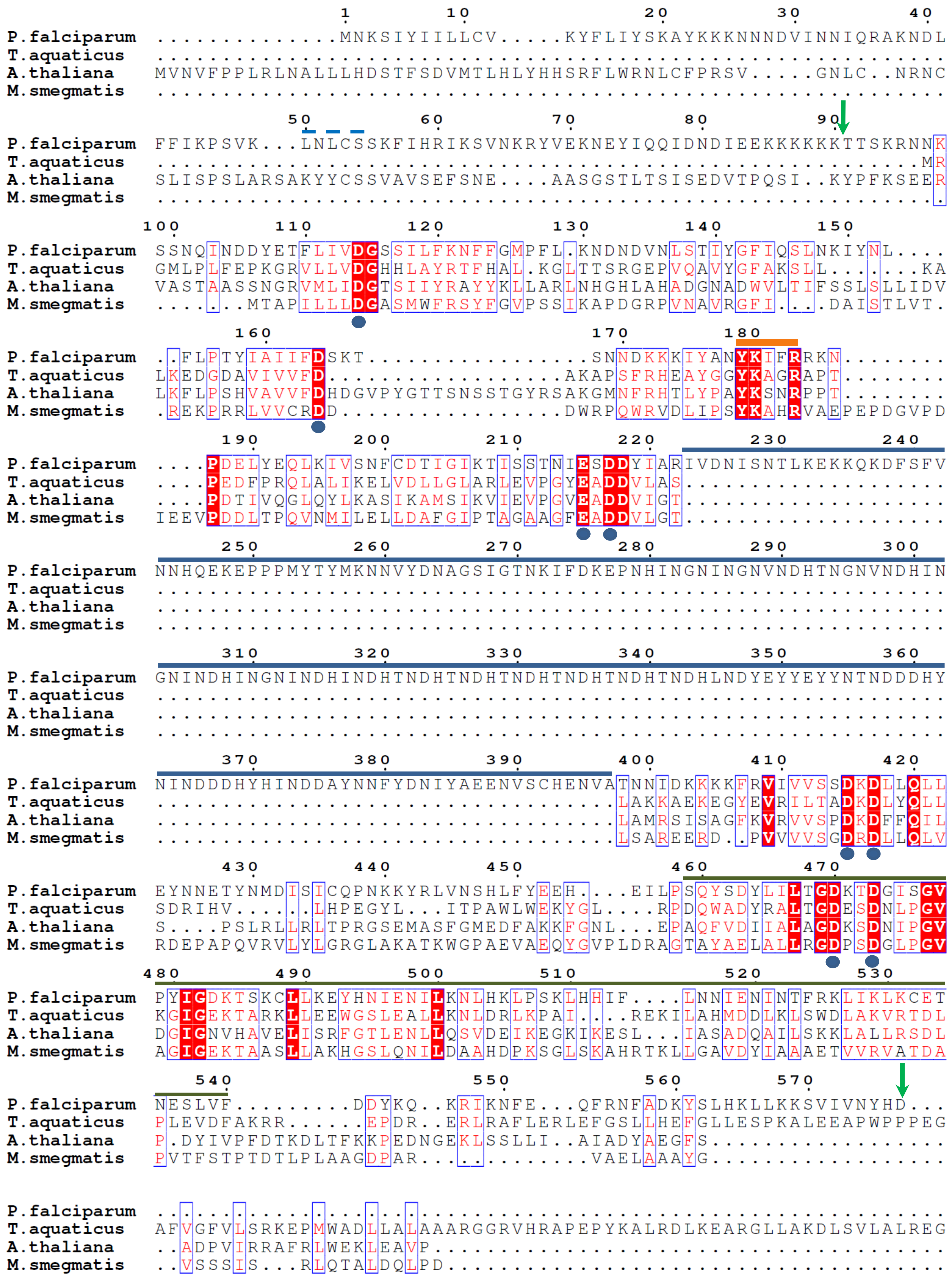

SI Figure S1. ClustalW alignment of *Pf*Exo with homolog from *Arabidopsis thaliana*, FenA of *Mycobacterium smegmatis* and the N-terminal 5'-3' exonuclease domain of Taq polymerase. Green line indicates the H3TH domain, the orange line marks the short loop which replaces the helical arch found in T5FEN, human FEN1 and EXO1. *M. smegmatis* FenA active site residues are marked by blue circles. The blue dashed line is the possible transit peptide cleavage site. Green arrows mark the start and stop positions of recombinant *Pf*Exo.
