# Supplementary material for: A Plasmodium apicoplast-targeted unique exonuclease/FEN exhibits interspecies functional differences attributable to an insertion that alters DNA-binding
Source: Nucleic Acids Res. 2024 Jun 18;52(13):7843–62. doi: 10.1093/nar/gkae512 (PMC11260460; doi:10.1093/nar/gkae512)
Supplement: gkae512_Supplemental_Files [file gkae512_supplemental_files.zip › Supplementary Figure S2.pdf]

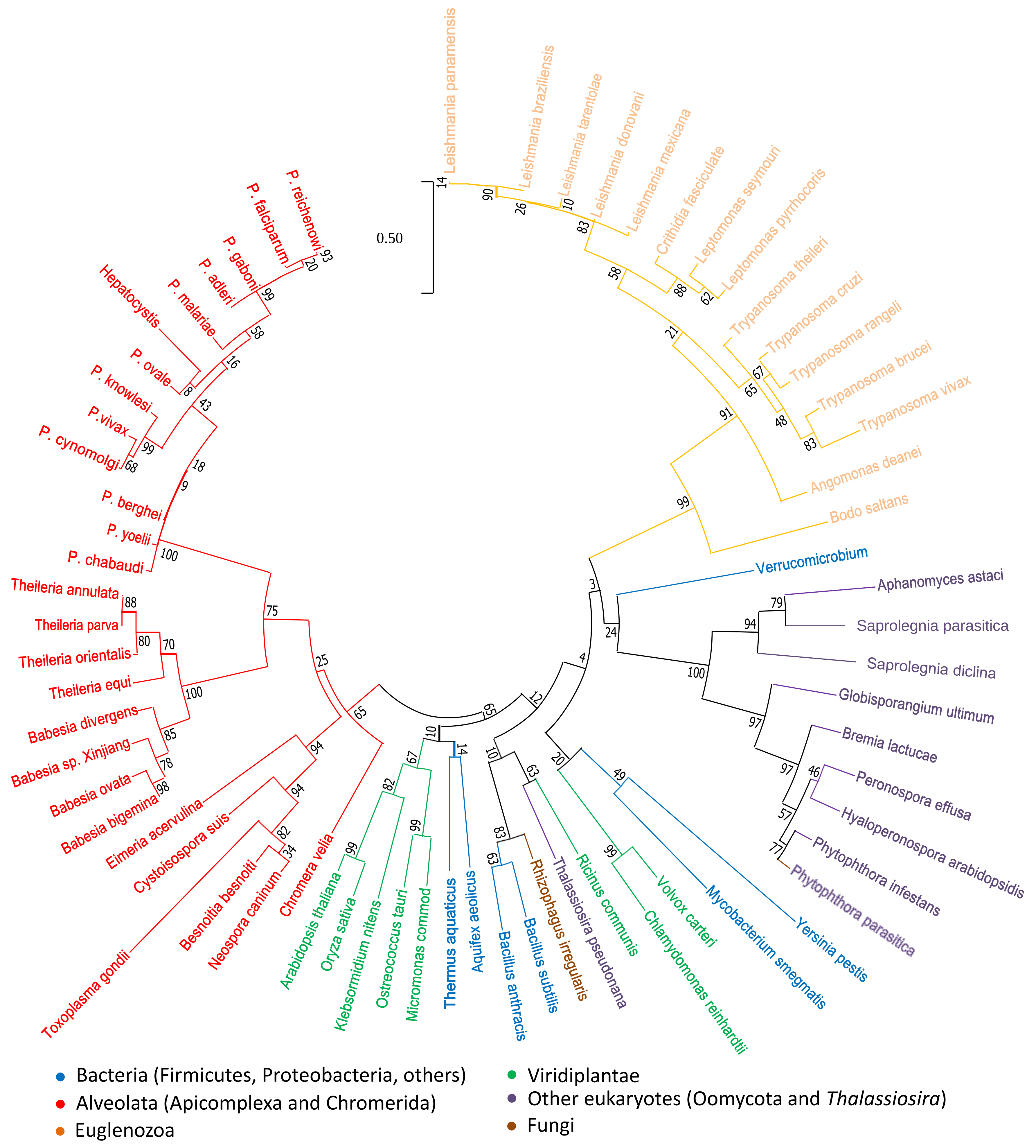

SI Figure S2. Phylogenetic analysis of 5'-3' exonuclease domain-containing proteins from Alveolates, Bacteria, Euglenozoa, Viridiplantae, Fungi, and other eukaryotes.
