# Supplementary material for: A Plasmodium apicoplast-targeted unique exonuclease/FEN exhibits interspecies functional differences attributable to an insertion that alters DNA-binding
Source: Nucleic Acids Res. 2024 Jun 18;52(13):7843–62. doi: 10.1093/nar/gkae512 (PMC11260460; doi:10.1093/nar/gkae512)
Supplement: gkae512_Supplemental_Files [file gkae512_supplemental_files.zip › Supplementary Figure S3.pdf]

A

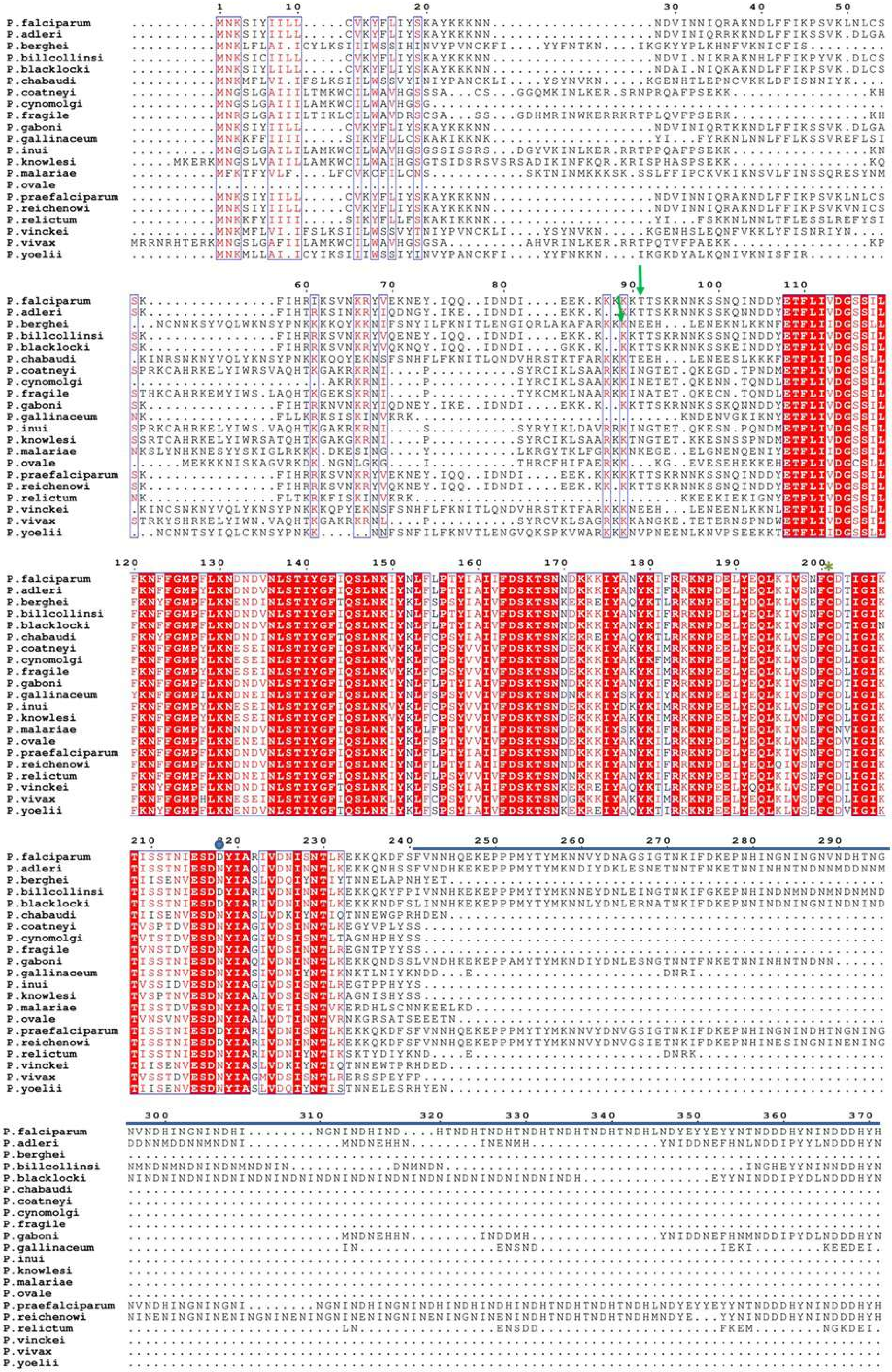
P.falciparum
P.adleri
P.berghei
P.billcollinsi
P.blacklocki
P.chabaudi
P.coatneyi
P.cynomolgi
P.fragile
P.gaboni
P.gallinaceum
P.inui
P.knowlesi
P.malariae
P.ovale
P.praefalciparum
P.reichenowi
P.relictum
P.vinckei
P.vivax
P.yoelii

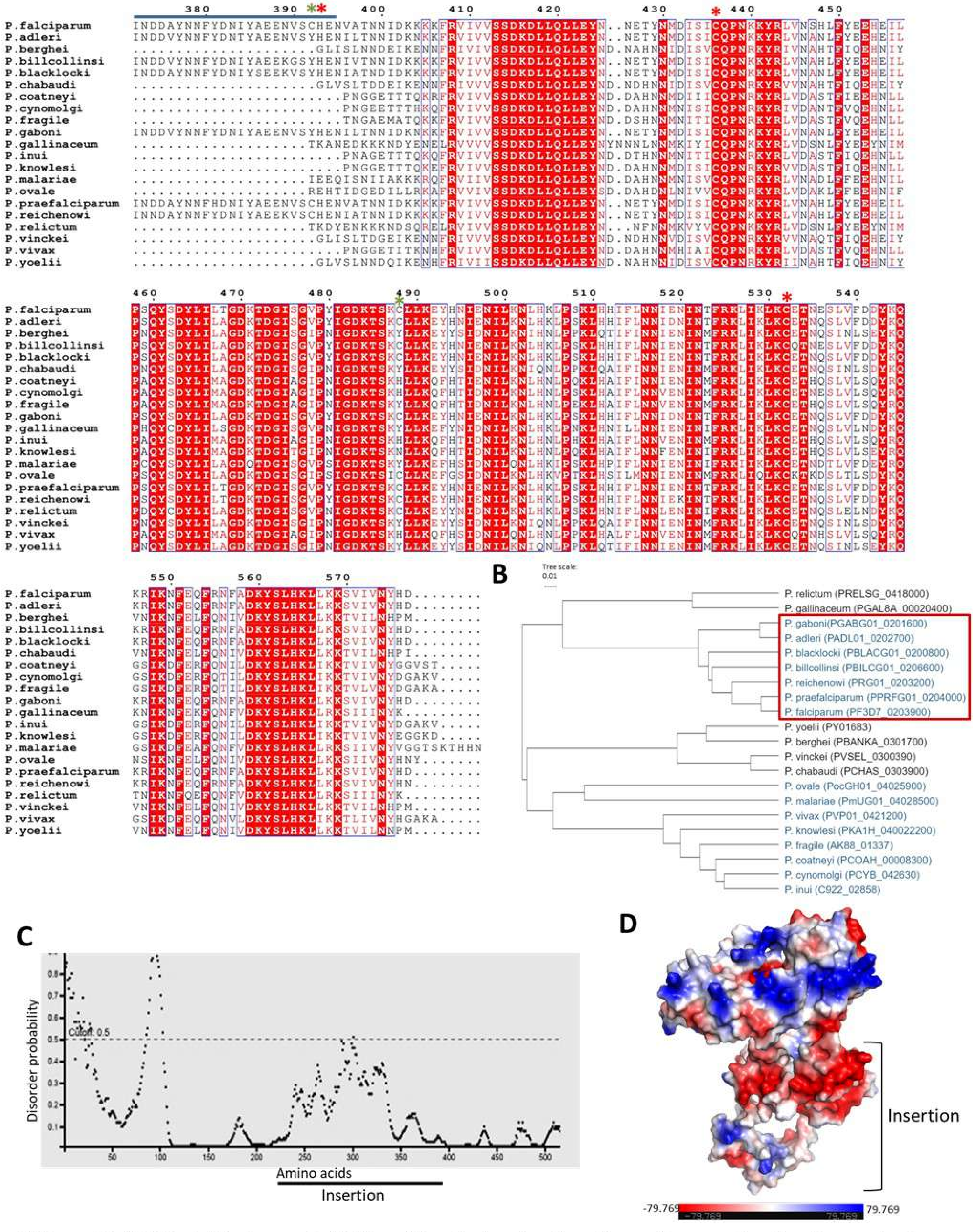

SI Figure S3. (**A**) ClustalW alignment of *Pf*Exo with orthologs in other *Plasmodium* species. Blue line marks the insertion sequence (LCR) in some primate-infecting *Plasmodium* species. Stars indicate Cys and His residues that were mutated in *Pf*Exo (red stars mark residues essential for [4Fe-4S] cluster binding). Blue circle marks the conserved Asp residue in *P. falciparum* and *P. reichenowi* which is replaced by Asn in other species. Green arrows mark the first amino acid of recombinant *Pf*Exo and *Pb*Exo (**B**) Phylogeny of *Pf*Exo homologs in *Plasmodium* species. Primate-infecting *Plasmodium* species are in blue; members of the subgenus Laverania (boxed in red) that carry an insertion cluster separately from the rest. (**C**) Protein disorder prediction for *Pf*Exo using Disopred software. (**D**) Charge distribution (generated in PyMOL) on modelled *Pf*Exo shown in Figure 1A.
