# Supplementary material for: A Plasmodium apicoplast-targeted unique exonuclease/FEN exhibits interspecies functional differences attributable to an insertion that alters DNA-binding
Source: Nucleic Acids Res. 2024 Jun 18;52(13):7843–62. doi: 10.1093/nar/gkae512 (PMC11260460; doi:10.1093/nar/gkae512)
Supplement: gkae512_Supplemental_Files [file gkae512_supplemental_files.zip › Supplementary Figures S4-S6.pdf]

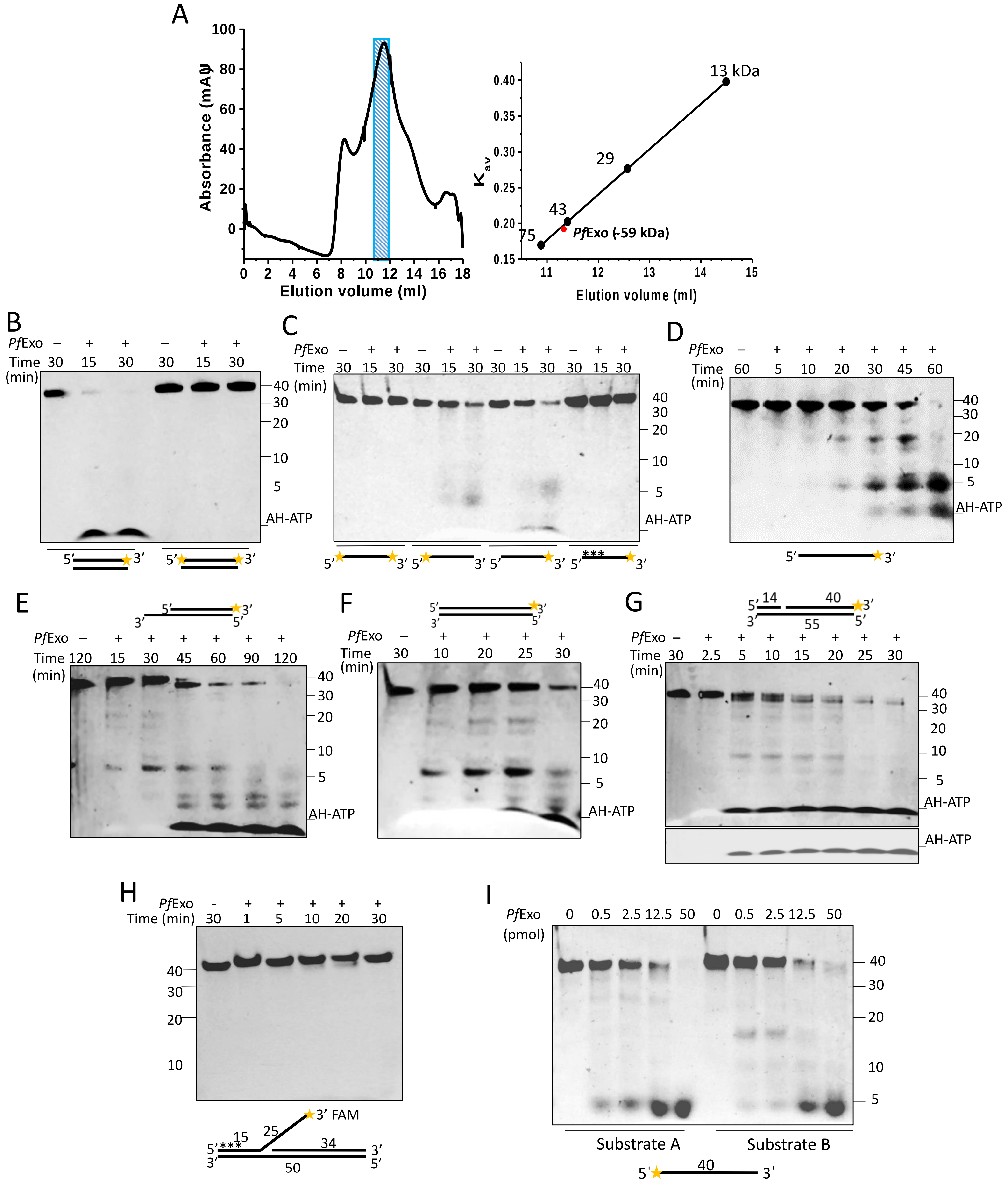

SI Figure S4. Purification and activity assays of *Pf*Exo. (**A**) Size exclusion chromatography of affinity-purified protein on S-75 column with the corresponding plot for molecular weight standards on the right. The blue shaded peak area yielded pure *Pf*Exo which was checked by SDS-PAGE (main Fig. 1B). (**B**) *Pf*Exo does not cleave blunt-end dsDNA blocked at both ends by conjugated FAM. (**C**) 3′-FAM labeled ssDNA blocked at the 5′-end by either FAM or phosphorothioate bonds (denoted by *) cannot be cleaved by *Pf*Exo. *Pf*Exo activity on DNA substrates at suboptimal conditions (100 mM NaCl, 20 °C) for lowering exonuclease processivity detects cleavage intermediates for 3′-FAM labeled ssDNA (**D**), and 5′-recessed (**E**), blunt-end (**F**) and 1 nt-gapped (**G**) dsDNA substrates. The bottom panel in (G) is a lower exposure of the gel showing accumulation of the terminal product over time. (**H**) *Pf*Exo does not cleave 3′-flap substrate. Asterisks denote three consecutive phosphorothioate bonds. (**I**) *Pf*Exo concentration-dependent 3′-5′ exonuclease activity on ssDNA with different AT/GC compositions (substrate A: 32% G+C; substrate B: 47.5 % G+C).

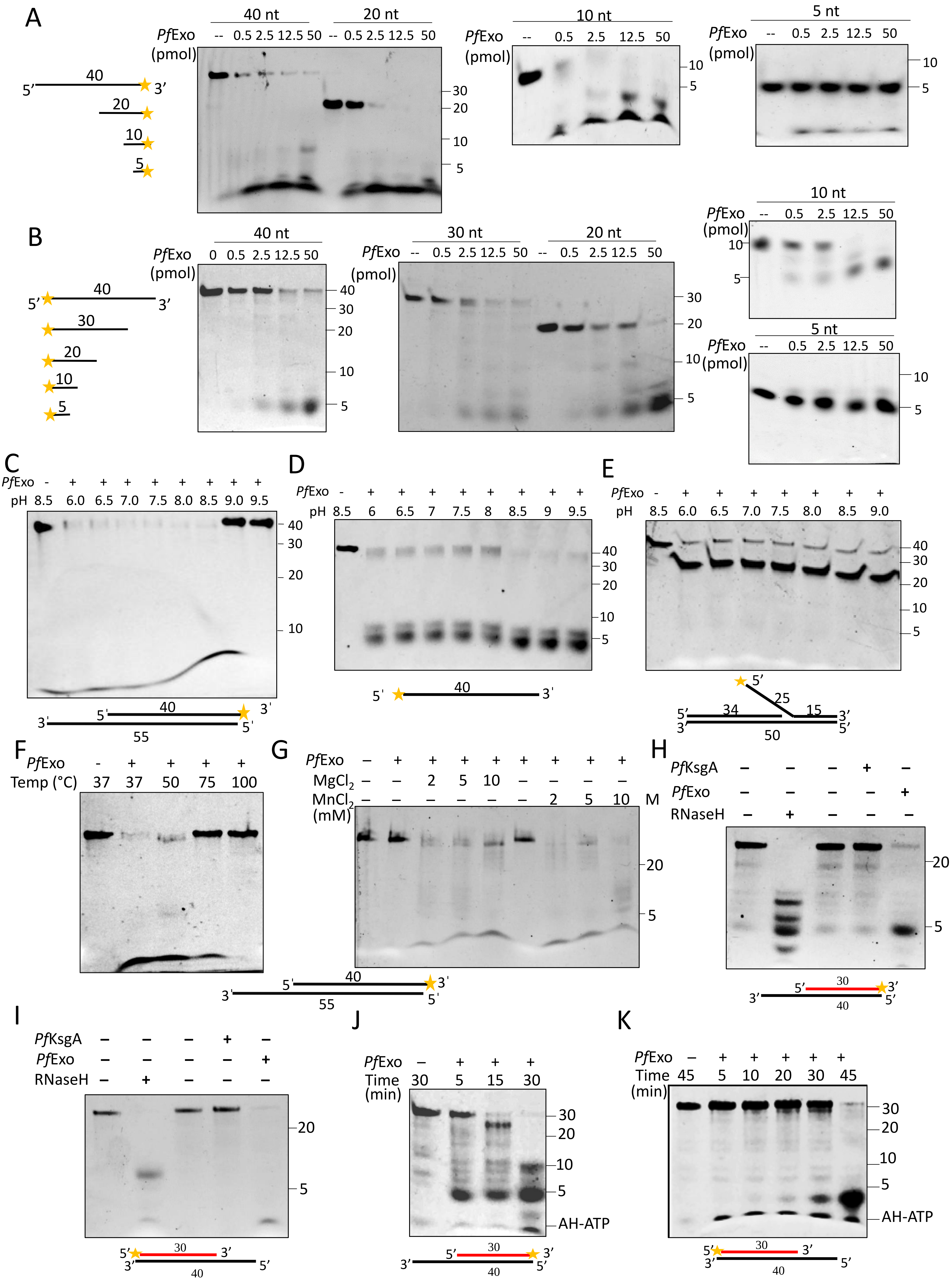

SI Figure S5. Substrate length, pH-, temperature- and metal ion-dependent activities of *Pf*Exo. Substrate size dependence for 5′-3′ exonuclease activity on ssDNA (**A**), and 3′-5′ exonuclease activity on ssDNA (**B**). pH dependent activity of *Pf*Exo on (**C**) 5′-recessed dsDNA, (**D**) 3′-5′ exonuclease on ssDNA, and (**E**) cleavage of 5′-flap substrate. The flap cleavage reaction was incubated for 10 min to limit exonuclease digestion of the flap. Temperature- (**F**) and metal ion- (**G**) dependent 5′-3′ exonuclease activity of *Pf*Exo. (**H, I**) RNase activity of *Pf*Exo. *Pf*KsgA (purified identically to *Pf*Exo) and RNaseH were used as negative and positive controls, respectively in the RNase assay on RNA-DNA hybrids. (**J, K**) Time-dependent cleavage of RNA-DNA hybrids in both polarities under suboptimal conditions (100 mM NaCl, 20 °C) for lowering *Pf*Exo processivity.

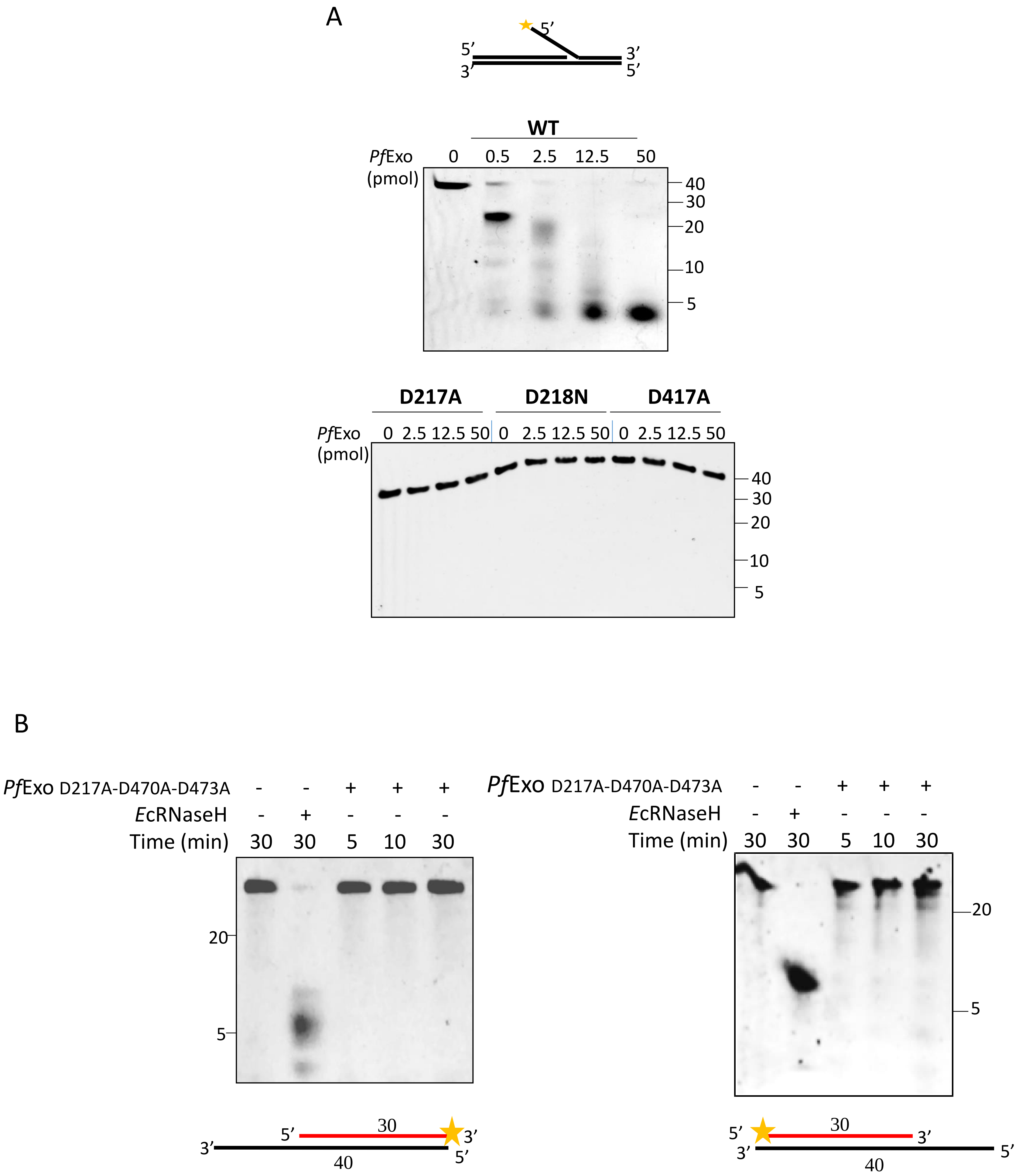

SI Figure S6. (**A**) Loss of 5' Flap endonuclease activity in single-site aspartate mutants of *Pf*Exo. (**B**) Loss of RNase activity of *Pf*ExoD217A-D470A-D473A on RNA-DNA hybrids in either polarity. *E. coli* RNaseH served as positive control.
