# Supplementary material for: A Plasmodium apicoplast-targeted unique exonuclease/FEN exhibits interspecies functional differences attributable to an insertion that alters DNA-binding
Source: Nucleic Acids Res. 2024 Jun 18;52(13):7843–62. doi: 10.1093/nar/gkae512 (PMC11260460; doi:10.1093/nar/gkae512)
Supplement: gkae512_Supplemental_Files [file gkae512_supplemental_files.zip › Supplementary Figures S7-S13.pdf]

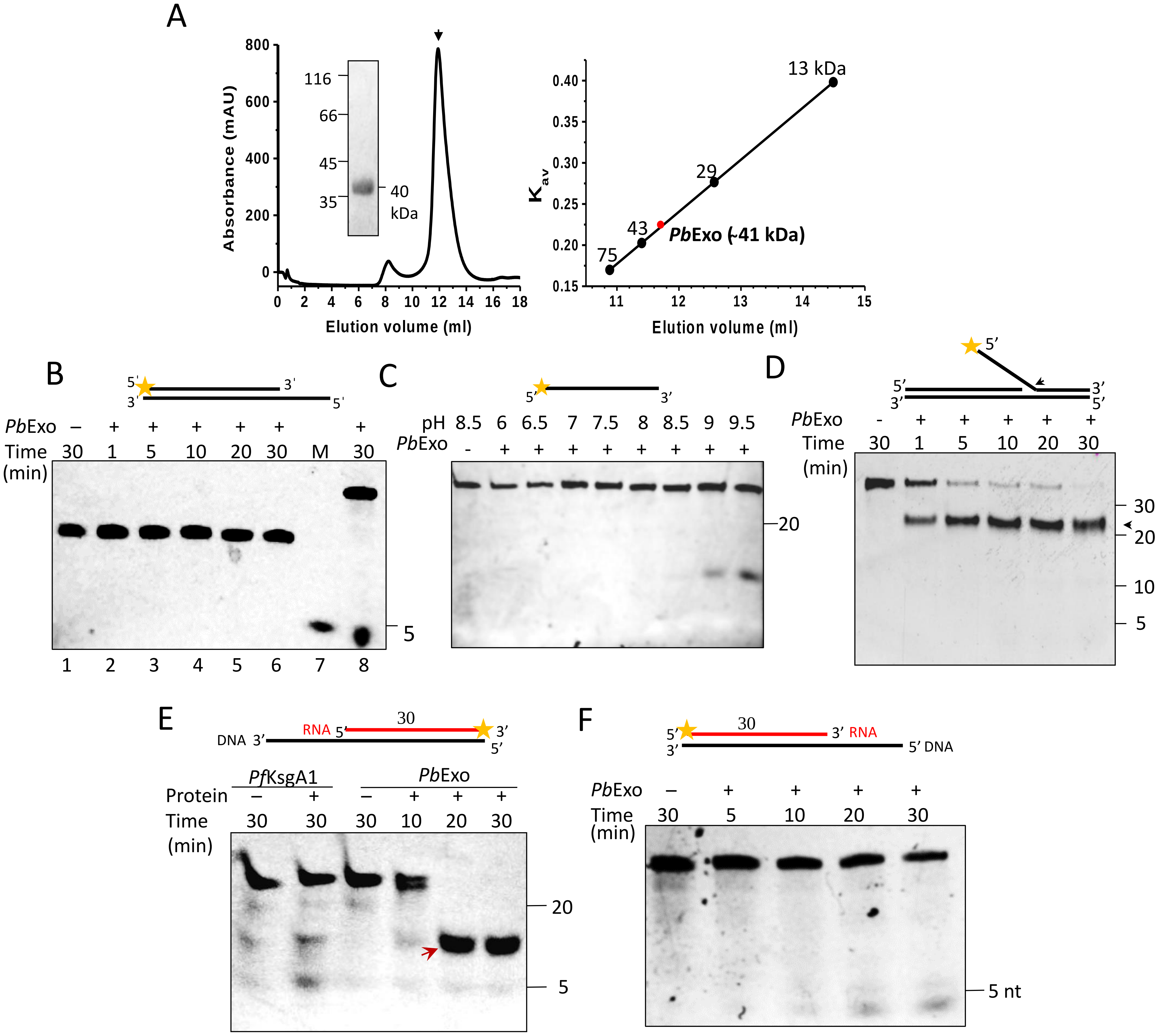

SI Figure S7. *Pb*Exo purification and activities. (**A**) Elution profile of *Pb*Exo upon size-exclusion chromatography on S-75 column and corresponding plot for chromatography molecular weight standards. Inset shows coomassie-stained SDS-PAGE of the purified protein. (**B**) *Pb*Exo does not cleave 3′-recessed dsDNA. 3′-5′ cleavage of ssDNA by *Pb*Exo (lanes 7-8) served as positive control for protein activity. (**C**) pH-dependent 3′-5′ cleavage of ssDNA by *Pb*Exo. (**D**) 5′-flap cleavage by *Pb*Exo. Arrow marks the ~25 nt cleavage product. *Pb*Exo cleaves RNA-DNA hybrid in both directions (**E** and **F**), with lower efficiency in the 3′-5′ direction (**F**). Identically purified *Pf*KsgA1 served as negative control in **E** suggesting that RNase cleavage was not due to a contaminating protein. Red arrow indicates the ~12 nt terminal RNA cleavage product.

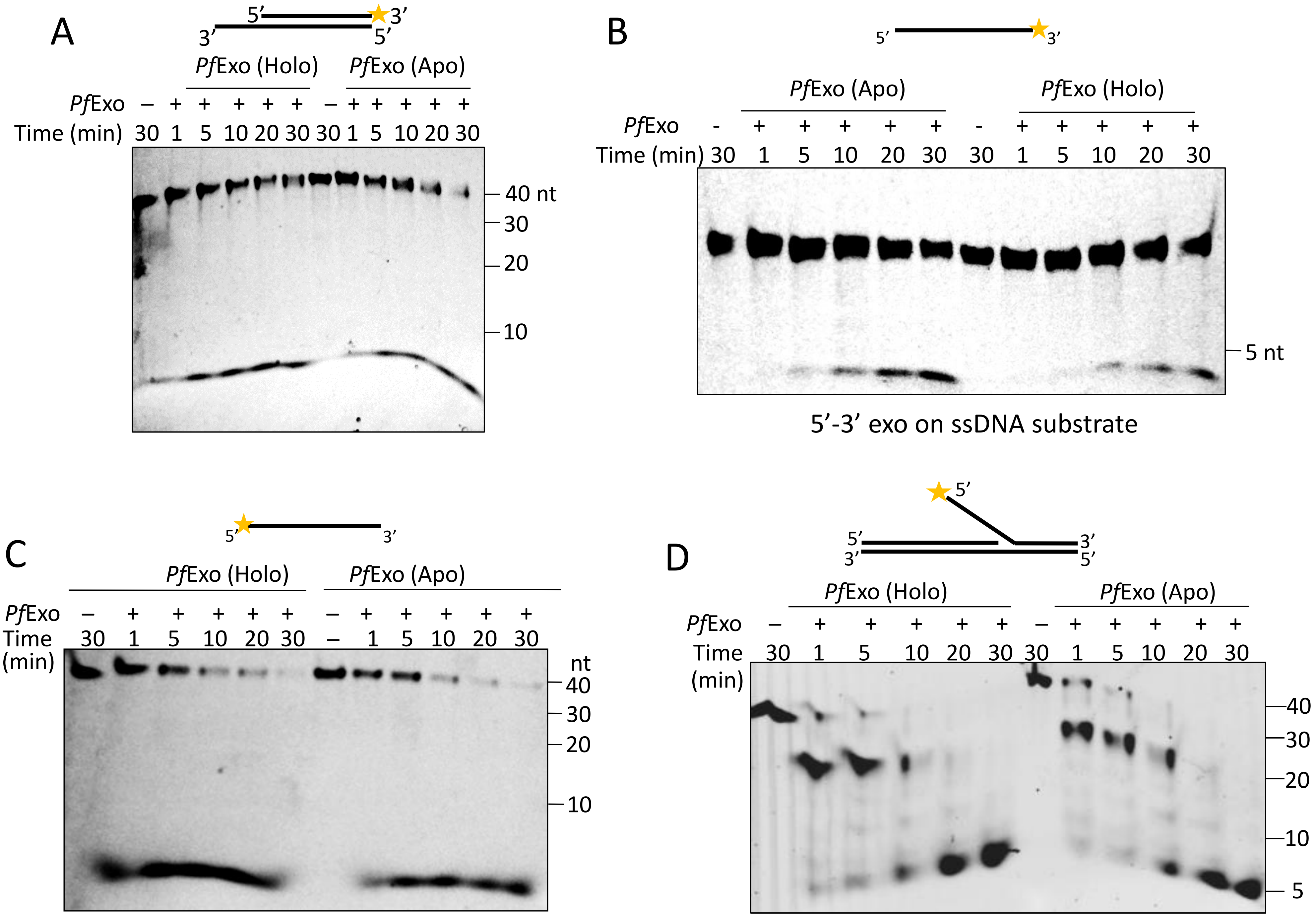

SI Figure S8. [Fe-S] cluster does not alter catalytic properties of *Pf*Exo in vitro. Comparison of exonuclease activity of *Pf*Exo holo- and apo-forms on 5′-recessed DNA (**A**), 5′-3′ exonuclease activity on ssDNA (**B**), 3′-5′ exonuclease activity on ssDNA (**C**) and endonucleolytic cleavage of 5′-flap substrate (**D**). All reactions were carried out in an anaerobic chamber.

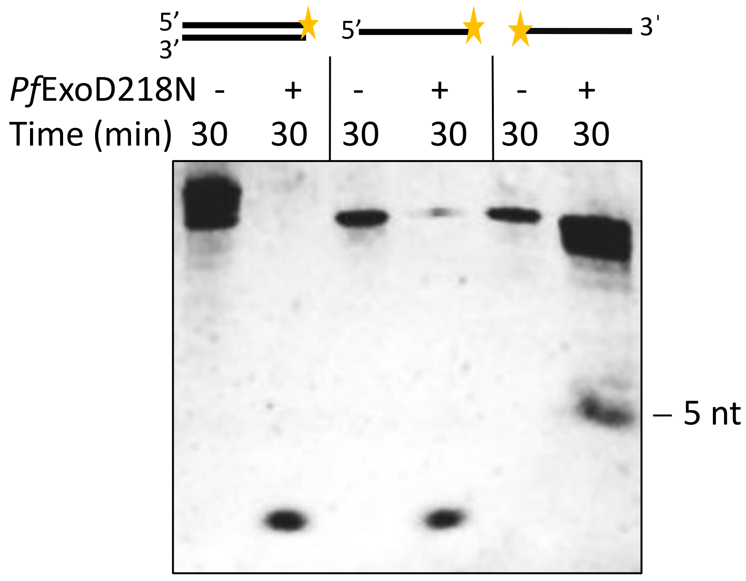

SI Figure S9. *PfE*xoD218N retains 5′-3′ exonuclease activity on blunt-end DNA, and 5′-3′ and 3′-5′ exonuclease activity on ssDNA.

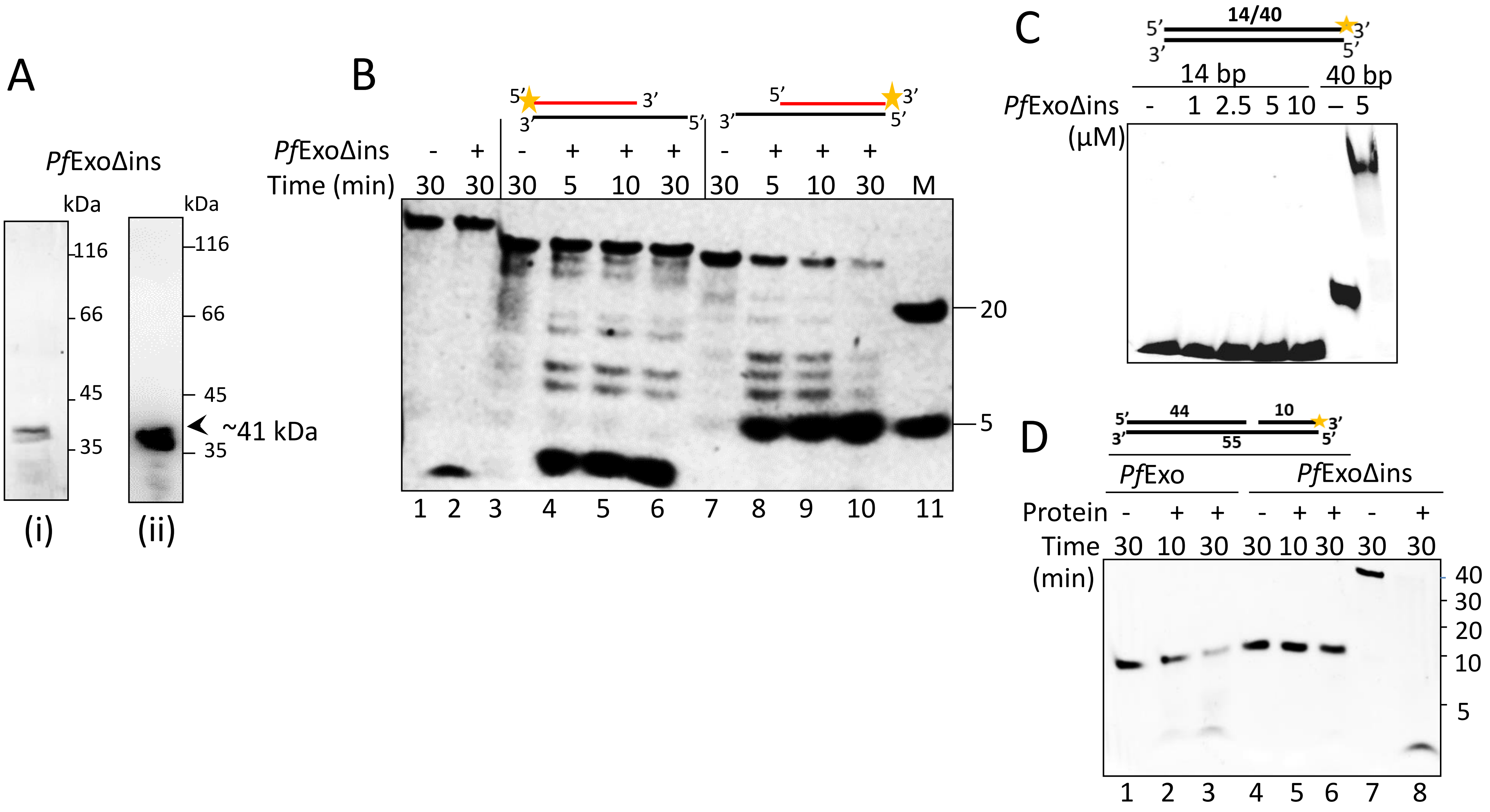

SI Figure S10. Purified *Pf*ExoΔins and its activity. (**A**) Coomassie-stained gel of purified *Pf*ExoΔins (molecular weight ~41 kDa) (i) and western blot of purified protein with anti-6X His Ab (ii). (**B**) RNase activity of *Pf*ExoΔins on RNA-DNA hybrids. Lanes 1-2 are positive controls for protein activity on 5′-recessed dsDNA. M, marker. (**C**) EMSA to detect binding of *Pf*ExoΔins with 14 bp or 40 bp blunt-end DNA substrates. (**D**) Activity of *Pf*Exo and *Pf*ExoΔins on a 1 nt-gapped substrate, with the gap positioned 44 nt from the 5′ end. Cleavage of 5'-recessed DNA by *Pf*ExoΔins (lanes 7-8) served as control for enzyme activity.

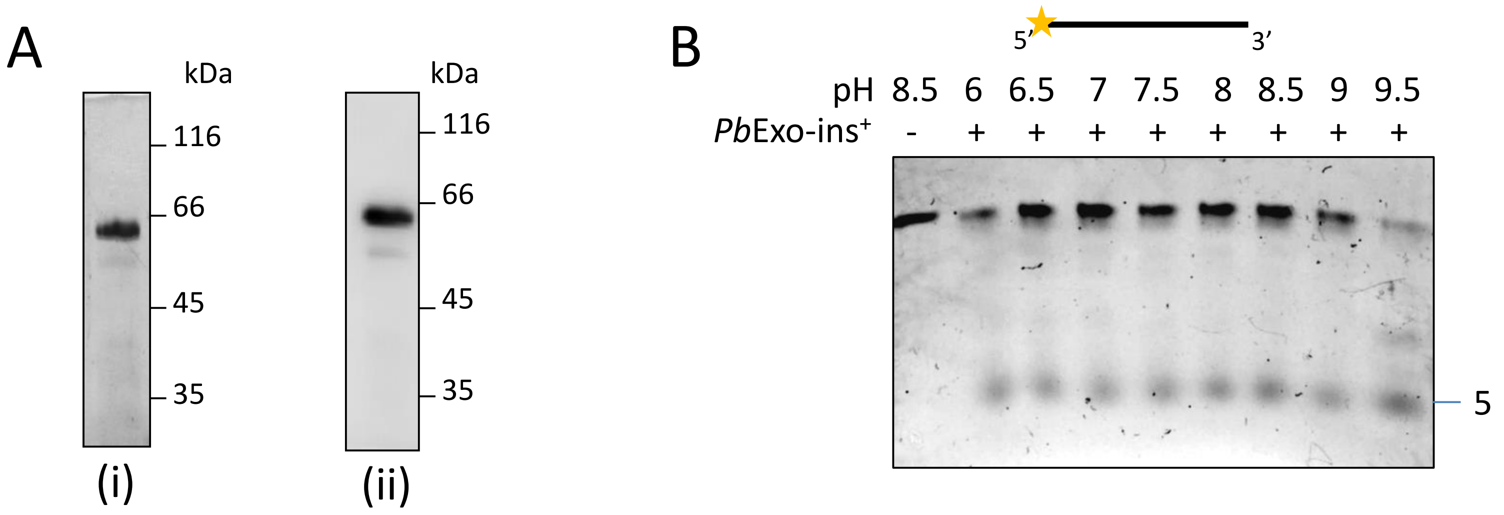

SI Figure S11. Purified *Pb*Exo-ins⁺ and activity. (**A**) Coomassie-stained gel of purified *Pb*Exo-ins⁺ (molecular weight ~59 kDa) (i) and western blot of purified protein with anti-6X His Ab (ii). (**B**) 3′-5′ exonuclease activity of *Pb*Exo-ins⁺ on ssDNA at different pH.

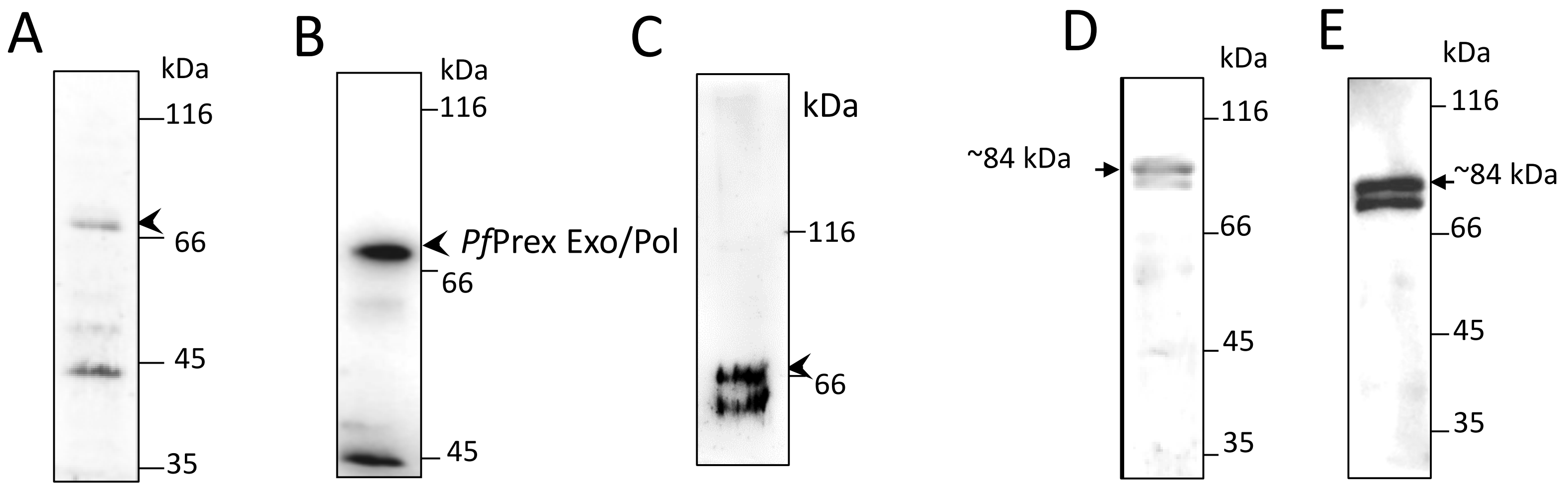

SI Figure S12. Recombinant *Pf*Prex Exo/Pol and GST-*Pf*Exo. (**A**) Coomassie-stained gel of purified *Pf*Prex Exo/Pol. (**B**) Western blot of purified *Pf*Prex Exo/Pol using anti-6X His Ab. (**C**) Western blot of *P. falciparum* lysate probed with anti-*Pf*Prex Exo/Pol sera detects a band close to the expected size of the *Pf*Prex Exo/Pol domain without the N-ter linker (~70 kDa) and a lower degraded/cleaved product. (**D**) Coomassie-stained SDS-PA gel of purified GST-*Pf*Exo. (**E**) Western blot of purified GST-*Pf*Exo with anti-GST Ab.

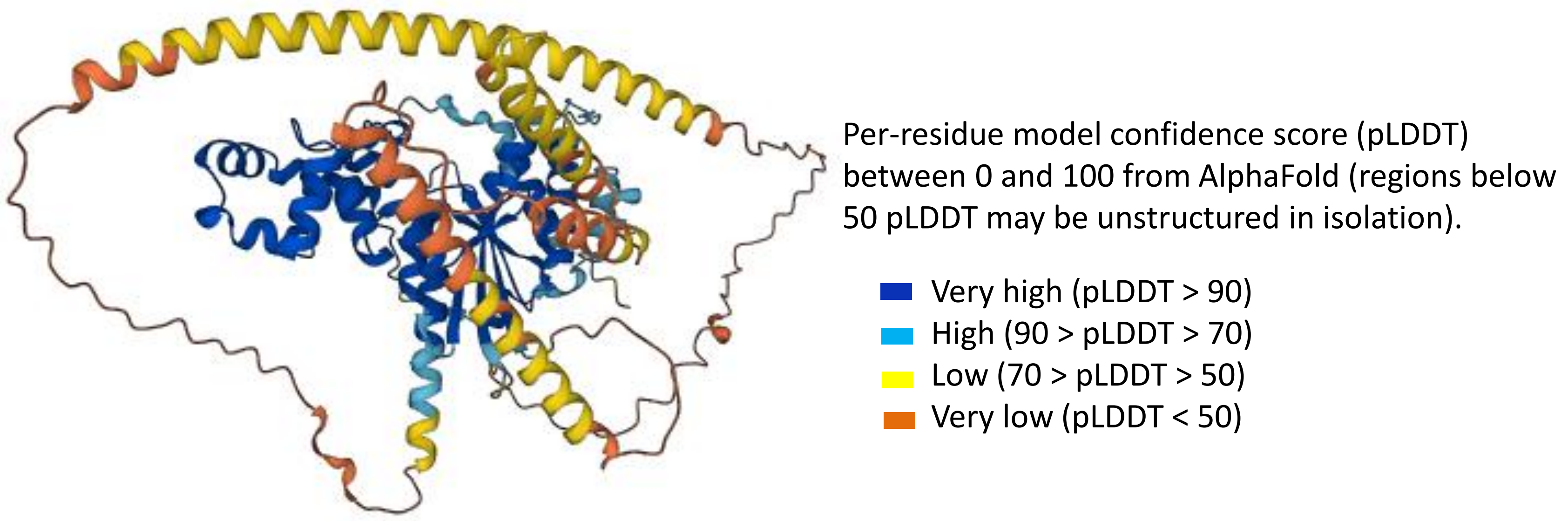

SI Figure S13. (**A**) Predicted *Pf*Exo structure taken from AlphaFold (PDB: AF-O96139-F1-model_v4; https://alphafold.ebi.ac.uk/) (15). The insertion sequence has very low pLDDT scores.
