# Supplementary material for: A Plasmodium apicoplast-targeted unique exonuclease/FEN exhibits interspecies functional differences attributable to an insertion that alters DNA-binding
Source: Nucleic Acids Res. 2024 Jun 18;52(13):7843–62. doi: 10.1093/nar/gkae512 (PMC11260460; doi:10.1093/nar/gkae512)
Supplement: gkae512_Supplemental_Files [file gkae512_supplemental_files.zip › Supplementary Tables.pdf]

**SI Table S1**. Comparison of functionalities of *Pf*Exo with known exonucleases that cleave both ssDNA and dsDNA substrates or have bipolar directionality on either substrate.

| ENZYME CLASS | ORGANISM | GENE | SUBSTRATE | POLARITY | PRODUCT | MECHANISM OF ACTION | FUNCTIONAL ATTRIBUTES | PATHWAY |
|---|---|---|---|---|---|---|---|---|
| **Eukaryotic Exonucleases** | | | | | | | | |
| **Exonuclease I** | *A. thaliana*<br>*H. sapiens* | EXOI | dsDNA<br><br>ssDNA<br><br>RNaseH (Okazaki fragment cleavage) | 5’-3’<br>3’-5’ (cryptic)<br>5’-3’ (v. low)<br><br>5’-3’ | Mono-di-tri nt | Processive | • 5’-3’ exo<br>• 5’Flap endo (preference for shorter flaps <5 nt) | DNA replication, MMR, DSBR, telomere maintenance (SI Ref. 1-3) |
| **Exonuclease V** | *A. thaliana*<br>*H. sapiens* | EXO5<br>*[4Fe-4S] | ssDNA | 5’-3’<br>3’-5’ | Di-nt | Processive | • 5’-3’ exo<br>• 3’-5’ exo | DSBR, replication fork restart (SI Ref.4-5) |
| **Bacterial Exonucleases** | | | | | | | | |
| **Exonuclease II** | *E. coli* | POLA | dsDNA, ssDNA | 3’-5’ | Mono-nt | Distributive | • Proofreading 3’-5’ exo | DNA repair (SI Ref.6-7) |
| **Exonuclease V** | *E. coli* | RECB,<br>RECC,<br>RECD | ssDNA<br>dsDNA | 3’-5’(on ssDNA)<br>5’-3’ (on dsDNA) | Oligo-nt (4-12) | Processive | • 3’-5’ exo<br>• 5’-3’ exo<br>• Bipolar helicase | DSB (SI Ref. 8-9) |
| **Exonuclease VII** | *E. coli* | XSEA<br>XSEB | ssDNA | 5’-3’<br>3’-5’ | Oligo-nt (4-12) | Processive | • 5’-3’ exo<br>• 3’-5’ exo | MMR, NIR (SI Ref. 10-11) |
| **Exonuclease X** | *E. coli* | EXOX | ssDNA, dsDNA | 3’-5’ | ? | Distributive | • 3’-5’ exo | MMR (SI Ref. 12) |
| **Bacteriophage Exonuclease** | | | | | | | | |
| **T5 Exonuclease** | Bacteriophage T5 | D15 | ssDNA, dsDNA | 5’-3’ | Mono-di-tri, oligo nt | Processive | • 5’-3’ exo<br>• 5’ Flap | DNA replication/repair (SI Ref.13-14) |
| ***Pf* 5’-3’ exonuclease** | | | | | | | | |
| ***Pf*Exo** | *P. falciparum* | PF3D7_0203900<br>*[4Fe-4S] | ssDNA, dsDNA, RNA-DNA hybrid | 5’-3’ (dsDNA, ssDNA)<br>3’-5’ (ssDNA),<br>Bipolar (RNA and RNA-DNA hybrid) | Mono-nt, 5-nt | Processive | • 5’-3’ exo<br>• 5’Flap endo<br>• 3’-5’ exo<br>• RNaseH | Replication/BER/MMR? |

**SI Table S2**. Primers for PCR amplification of *Pf*Prex, *Pf*Exo, *Pb*Exo and cloning of *Pf*ExoΔins

| Protein | Primer | Sequence |
|---|---|---|
| ***Pf*Prex Exo/Pol domain** | Forward | 5'-CGCGGATCCTATACAATGGTAAAAAATGGTAAC-3' |
| | Reverse | 5'-CGCGTCGACATCCTTTGATCCCCAATTTTC-3' |
| ***Pf*Exo** | Forward (also primer 'a' for *Pf*ExoΔins) | 5'-CGCGGATCCACTACATCTAAACGCAACAAT-3' |
| | Reverse (also primer 'd' for *Pf*ExoΔins) | 5'-CGCGTCGACATCATGGTAATTAACTAT-3' |
| ***Pb*Exo** | Forward | 5'-CGCGGATCCAAAAACGAAGAACACTTAGAAAAT-3' |
| | Reverse | 5'-CGCAAGCTTCATGGGATGATTCAATATAACGGT-3' |
| ***Pf*ExoΔins** | b | 5'-TGCAACATTTTCGGAAAAATCCTTTTGTTTCTTTTC-3' |
| | c | 5'-AAGGATTTTTCCGAGAATGTTGCAACAAATAATATAG-3' |

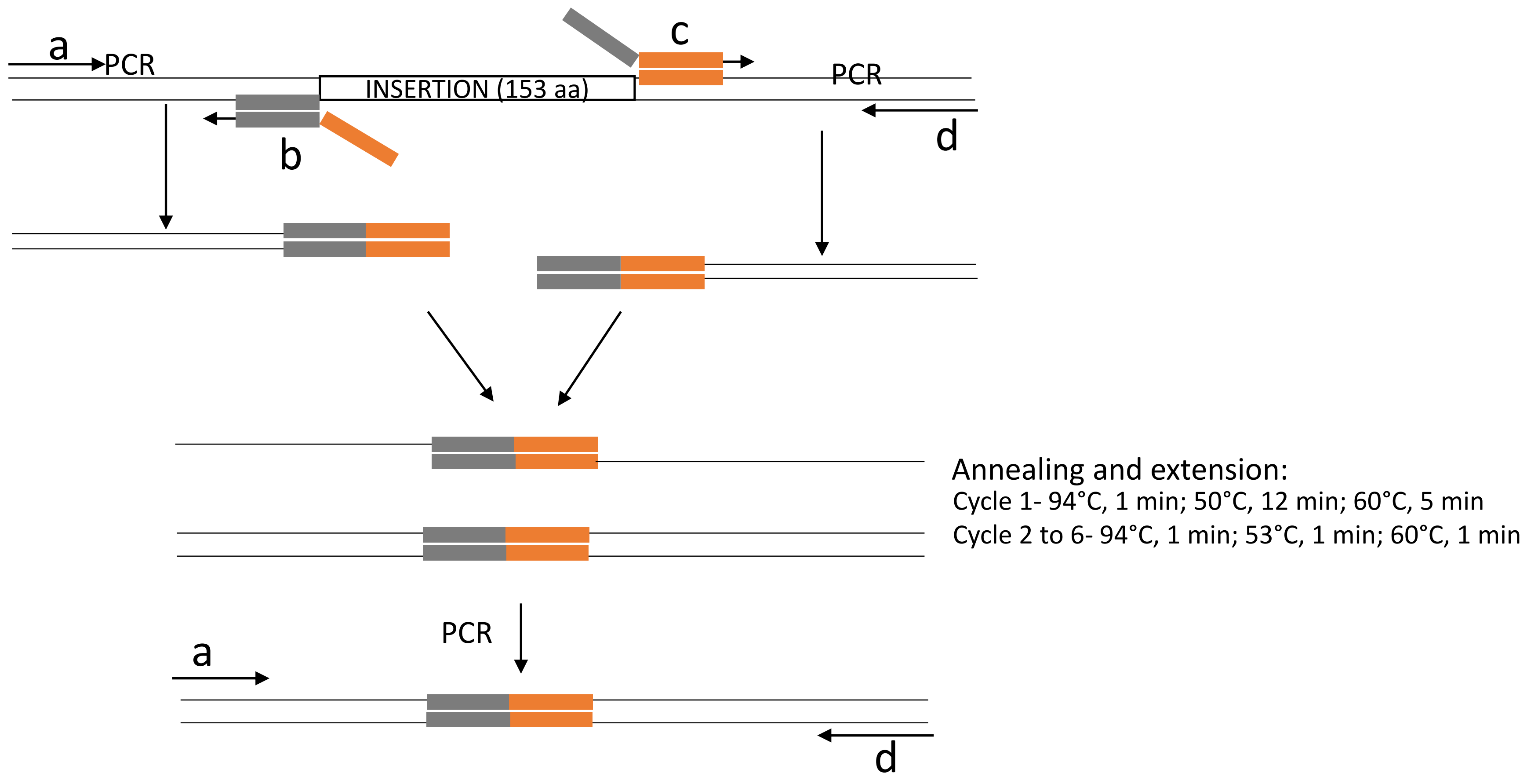


*Restriction enzyme sites are underlined*

**SI Table S3**. Substrates used in nuclease and protein binding assays. * indicates phosphorothioate bond.

| **Activity** (exo/endo-nuclease) | **Substrate** | **Sequence** | **Schematic** |
|---|---|---|---|
| **dsDNA, 5'-3' exo** | 5'-recessed | 5'-AAAACGTCAACTAGCTTCTCAAACACCTCATATATCTTAA/**6-FAM**/-3'<br>3'-GATCACATGTACGTATTTTGCAGTTGATCGAAGAGTTTGTGGAGTATATAGAATT-5' | 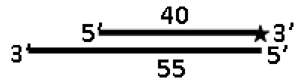 |
| | Blunt-end | 5'- AAAACGTCAACTAGCTTCTCAAACACCTCATATATCTTAA/**6-FAM**/-3'<br>3'-TTTTGCAGTTGATCGAAGAGTTTGTGGAGTATATAGAATT-5' | 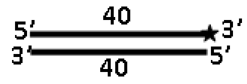 |
| | Blunt-end (14) | 5'- CTAGTGTACATGCA/**6-FAM**/-3'<br>3'- GATCACATGTACGT-5' | 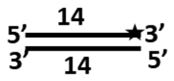 |
| | 1 nt-gapped (14 nt 5' of gap) | 5'- CTAGTGTACATGCA_AAAACGTCAACTAGCTTCTCAAACACCTCATATATCTTAA/**6-FAM**/-3<br>3'-GATCACATGTACGTATTTTGCAGTTGATCGAAGAGTTTGTGGAGTATATAGAATT-5' | 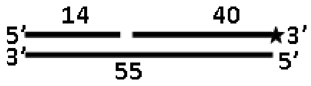 |
| | 1 nt-gapped (44 nt 5' of gap) | 5'- CTAGTGTACATGCATAAAACGTCAACTAGCTTCTCAAACACCTC_TATATCTTAA/**6-FAM**/-3'<br>3'-GATCACATGTACGTATTTTGCAGTTGATCGAAGAGTTTGTGGAGTATATAGAATT -5' | 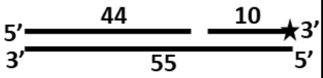 |
| **dsDNA, 3'-5' exo** | 3'-recessed | 5'-/**6FAM**/-AAAACGTCAACTAGCTTCTCAAACAGCTCATATATCTTAA-3'<br>3'-TTTTGCAGTTGATCGAAGAGTTTGTCGAGTATATAGAATTGATCACATG*T*A*C*G*T*A-5' | 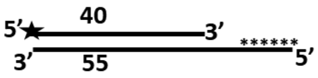 |
| | Blunt-end | 5'-/**6-FAM**/AAAACGTCAACTAGCTTCTCAAACAGCTCATATATCTTAA-3'<br>3'-TTTTGCAGTTGATCGAAGAGTTTGTCGAGTATAT*A*G*A*A*T*T-5' | 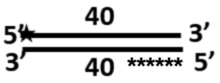 |
| **ssDNA, 5'-3' exo** | ssDNA, 3'FAM | 5'- AAAACGTCAACTAGCTTCTCAAACACCTCATATATCTTAA/**6-FAM**/-3'<br>5'- AAACACCTCATATATCTTAA/**6-FAM**/-3'<br>5'- TATATCTTAA/**6-FAM**/-3'<br>5'- CTTAA/**6-FAM**/-3' | 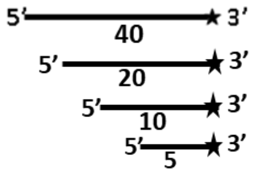 |
| **ssDNA 3'-5' exo** | ssDNA, 5'FAM | 5'-/**6-FAM**/AAAACGTCAACTAGCTTCTCAAACAGCTCATATATCTTAA-3'<br>5'-/**6-FAM**/AAAACGTCAACTAGCTTCTCAAACAGCTCA- 3'<br>5'-/**6-FAM**/AAAACGTCAACTAGCTTC- 3'<br>5'-/**6-FAM**/AAAACGTCAA- 3'<br>5'-/**6-FAM**/CTTAA- 3' | 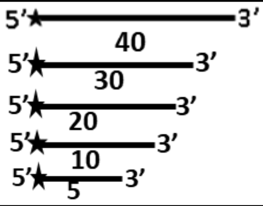 |
| **Terminal FAM cleavage** | Dual FAM-conjugated ssDNA and blunt-end dsDNA | 5'-/**6-FAM**/AAAACGTCAACTAGCTTCTCAAACACCTCATATATCTTAA/**6-FAM**/-3'<br><br>5'-/**6-FAM**/AAAACGTCAACTAGCTTCTCAAACACCTCATATATCTTAA/**6-FAM**/-3'<br>3'-TTTTGCAGTTGATCGAAGAGTTTGTGGAGTATATAGAATT-5' | 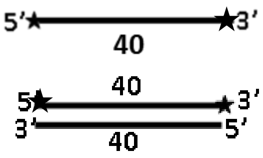 |
| **5'Flap$_{25}$, endo** | 5'FAM-flap | 5'-/**6-FAM**/-AAAACGTCAACTAGCTTCTCAAACA<br>5'-CAGTCCGAGCTAGGCAACATACGATAATCCATAC_GCTCATATATCTTAA-3'<br>3'-GTCAGGCTCGATCCGTTGTATGCTATTAGGTATGACGAGTATATAGAATT-5' | 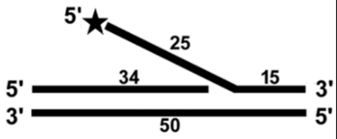 |
| **3'Flap$_{25}$, endo** | 3' FAM-flap | GAGCGAAGCGGGATCGCAGACTCAA-/**6FAM**/-3'<br>5'C*A*G*TCCGAGCTAGGC_ACATACGATAATCCATACAGTGAGCGAATACGAG-3'<br>3'-GTCAGGCTCGATCCGTTGTATGCTATTAGGTATGTCACTCGCTTATGCTC-5' | 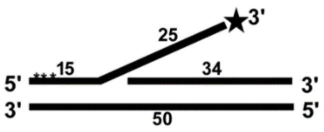 |
| **RNA-DNA hybrid, 5'-3' exo** | 5'-recessed | 5'-CUAGCUUCUCAAACACCUCAUAUAUCUUAA/**6-FAM**/-3'<br>3'–GATCACATGTACGTATTTTGCAGTTGATCGAAGAGTTTGTGGAGTATATAGAATT-5' | 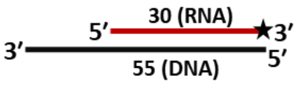 |
| **RNA-DNA hybrid, 3'-5' exo** | 3'-recessed | 5'-/**6-FAM**/-CUAGUGUACAUGCAUAAAACGUCAACUAGC-3'<br>3'-GATCACATGTACGTATTTTGCAGTTGATCGAAGAGTTTGTGGAGTATATAGAATT-5' | 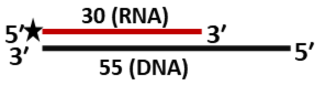 |
| **BLI template** | Blunt-end | 5'-/5Biosg/CAGTCCGAGCTAGGCAACATACGATAATCCATAC-3'<br>3'-GTCAGGCTCGATCCGTTGTATGCTATTAGGTATG-5' | 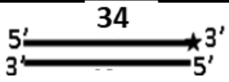 |

**SI Table S4**. Primers used to disrupt the *Pb*Exo gene.

| Primer ID | Sequence |
|---|---|
| 1430 | GACGTCGACCTTATTTATGTGTATGTACCTT |
| 1431 | GACGTCGACGGCCAAGAACAATTTATTCAT |
| 1432 | GCGGCCGCCATCCCATGTAGAGCCTAT |
| 1433 | GGCGCGCCGGAAAATTTTCTGAGCAAGTC |
| 1538 | ATTCAAGATACTCTCAAAACC |
| 1539 | CACACATTATATTGGTGTGC |
| 1215 | GTTGTCTCTTCAATGATTCATAAATAG |
| 1225 | TTCCGCAATTTGTTGTACATA |
| 1704 | GAAACTGGGCTAATTTCGTTAA |
| 1705 | CATGGGATGATTCAATATAACG |

*Restriction enzyme sites are underlined*

**SI Table S5:** Percent cleavage of mutants compared to wild type *Pf*Exo. Metal binding sites likely to be impacted by the mutations are in blue.

| Protein (metal binding site) | 5'-flap endonuclease activity | % exonuclease activity | | | |
|---|---|---|---|---|---|
| | | 5'-recessed dsDNA | 1 nt gapped dsDNA | Blunt-end dsDNA | 3'-5' ssDNA |
| **WT** | **+++** | **100** | **100** | **100** | **100** |
| **D218N (M1/M2)** | **-** | **86.8** | **ND** | **ND** | **73.4** |
| **D217A (M1/M2)** | **-** | **29.9** | **ND** | **ND** | **45.8** |
| **D417A (M2/M3)** | **-** | **41.4** | **ND** | **ND** | **91.7** |
| **D417A+D470A+D473A (M2/M3)** | **ND** | **26.0** | **≤0.1** | **≤0.1** | **98.1** |
| **D217A+D470A+D473A (M1/M2/M3)** | **ND** | **12.3** | **1.5** | **≤0.1** | **39.9** |

ND: not determined

## SI-References


1. Wilson,D.M., Carney,J.P., Coleman,M.A., Adamson,A.W., Christensen,M. and Lamerdin,J.E. (1998) Hex1: a new human Rad2 nuclease family member with homology to yeast exonuclease 1. *Nucleic Acids Res.*, **26**, 3762.
2. Dzantiev,L., Constantin,N., Genschel,J., Iyer,R.R., Burgers,P.M. and Modrich,P. (2004) A defined human system that supports bidirectional mismatch-provoked excision. *Mol. Cell*, **15**, 31–41.
3. Keijzers,G., Bakula,D., Petr,M.A., Madsen,N.G.K., Teklu,A., Mkrtchyan,G., Osborne,B. and Scheibye-Knudsen,M. (2019) Human exonuclease 1 (EXO1) regulatory functions in DNA replication with putative roles in cancer. *Int. J. Mol. Sci.*, **20**, 1–15.
4. Sparks,J.L., Kumar,R., Singhs,M., Wold,M.S., Pandita,T.K. and Burgers,P.M. (2012) Human exonuclease 5 is a novel sliding exonuclease required for genome stability. *J. Biol. Chem.*, **287**, 42773–42783.
5. Hambarde,S., Tsai,C.L., Pandita,R.K., Bacolla,A., Maitra,A., Charaka,V., Hunt,C.R., Kumar,R., Limbo,O., Le Meur,R., *et al.* (2021) EXO5-DNA structure and BLM interactions direct DNA resection critical for ATR-dependent replication restart. *Mol. Cell*, **81**, 2989-3006.e9.
6. Lehman,I.R. and Richardson,C.C. (1964) The Deoxyribonucleases of *Escherichia coli*: IV. An exonuclease activity present in purified preparations of deoxyribonucleic acid polymerase. *J. Biol. Chem.*, **239**, 233–241.
7. Setlow,P. and Kornberg,A. (1972) Deoxyribonucleic Acid Polymerase: Two distinct enzymes in one polypeptide. *J. Biol. Chem.*, **247**, 232–240.
8. Amundsen,S.K. and Smith,G.R. (2007) Chi hotspot activity in *Escherichia coli* without RecBCD Exonuclease activity: Implications for the mechanism of recombination. *Genetics*, **175**, 41–54.
9. Spies,M., Amitani,I., Baskin,R.J. and Kowalczykowski,S.C. (2007) RecBCD enzyme switches lead motor subunits in response to χ recognition. *Cell*, **131**, 694–705.
10. Chase,J.W. and Richardson,C.C. (1974) Exonuclease VII of *Escherichia coli*. *J. Biol. Chem.*, **249**, 4553–4561.
11. Vales,L.D., Rabin,B.A. and Chase,J.W. (1983) Isolation and preliminary characterization of *Escherichia coli* mutants deficient in exonuclease VII. *J. Bacteriol.*, **155**, 1116–1122.
12. Viswanathan,M. and Lovett,S.T. (1999) Exonuclease X of *Escherichia coli*. A novel 3'-5' DNase and Dnaq superfamily member involved in DNA repair. *J. Biol. Chem.*, **274**, 30094–30100.
13. Pickering,T.J., Garforth,S.J., Thorpe,S.J., Sayers,J.R. and Grasby,J.A. (1999) A single cleavage assay for T5 5'→3' exonuclease: Determination of the catalytic parameters for wild-type and mutant proteins. *Nucleic Acids Res.*, **27**, 730–735.
14. Desai,N.A. and Shankar,V. (2003) Single-strand-specific nucleases. *FEMS Microbiol. Rev.*, **26**, 457–491.
15. Jumper, J., Evans, R., Pritzel, A. et al. (2021) Highly accurate protein structure prediction with AlphaFold. *Nature* **596**, 583–589.
